# Achieving Phase Control of Polymorphic Tungsten Carbide Catalysts

**DOI:** 10.1021/acscatal.5c07774

**Published:** 2026-01-05

**Authors:** Sinhara M. H. D. Perera, Eva Ciuffetelli, Marc D. Porosoff

**Affiliations:** Department of Chemical and Sustainability Engineering, 6927 University of Rochester, Rochester, New York 14627, United States

**Keywords:** tungsten carbide, transition metal carbides, CO_2_ conversion, reverse water-gas shift, CO_2_ hydrogenation

## Abstract

The polymorphism of tungsten carbide (W_*x*_C) and the challenge of selectively synthesizing pure phases have impeded a precise understanding of catalytic structure–property relationships. This study establishes a framework for phase-selective synthesis of W_*x*_C through controlling carburization kinetics. By maintaining particle sizes below 10 nm, β-W_2_C is selectively synthesized using gaseous carbon precursors (CH_4_/H_2_) via temperature-programmed carburization (TPC). Our findings reveal that W_2_C stabilization is predominantly dictated by particle size and carburization kinetics rather than support interactions, providing a tunable approach to synthesize tungsten carbide catalysts. We elucidate the mechanistic pathway of WO_*x*_ carburization, demonstrating that CH_4_ activation occurs at mild temperatures via lattice oxygen. Our reactor studies establish *ex situ* synthesized β-W_2_C as an active and stable catalyst for the reverse water-gas shift (RWGS) reaction. However, the need for passivation and reduction pretreatment leads to a complex surface structure with diminished intrinsic activity. In contrast, our *in situ* synthesis protocol for β-W_2_C eliminates the need for passivation and exhibits increased CO STY during RWGS, illustrating the intrinsically higher activity compared to metallic W, WC_1–*x*_ (0.5 < *x* < 1), and stoichiometric WC.

## Graphical abstract

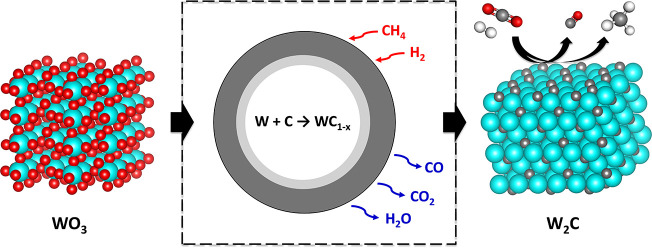

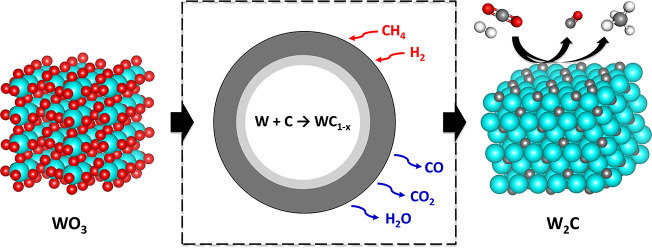

## 1 Introduction

Transition metal carbides, such as molybdenum carbide (Mo_*x*_C) and tungsten carbide (W_*x*_C), have been recognized as effective catalysts for CO_2_ hydrogenation,^1–5^ demonstrating comparable performance because of their similar electronic structures.^6,7^ However, the precise identification of the active phase(s) within W_*x*_C remains underexplored. Applications of W_*x*_C in heterogeneous catalysis first emerged in the 1970s when anodic hydrogen oxidation was benchmarked against platinum (Pt) electrodes.^8^ A few years later, the Pt-like behavior of W_*x*_C became evident with demonstrated activity in reactions typically exclusive to Pt.^9^ This breakthrough challenged conventional understanding, highlighting the unique electronic and structural properties of transition metal carbides, and in particular, W_*x*_C. Since then, W_*x*_C has been explored in several catalytic applications, including water splitting, electrooxidation, polysulfide conversion, biomass valorization, polyolefin hydrocracking and a range of isomerization, reforming, hydrogenolysis, and hydrogenation reactions.^10–18^

Despite numerous examples of W_*x*_C as an active and versatile catalyst, precise structure–property relationships are ambiguous due to complex polymorphism and oxyphilicity.^19,20^ W_*x*_C-based catalysts exist as heterogeneous multiphase mixtures, comprising various tungsten carbide and oxide phases, including W_2_C, WC, WC_1–*x*_ (0.5 < *x* < 1), metallic W, WO_3_, and substoichiometric WO_3–*x*_ (0 < *x* < 3).^21^ Exacerbating this issue, there are inconsistencies in the literature on W_*x*_C characterization and nomenclature, leading to uncertainty in identifying structure–activity relationships.^22,23^

W_*x*_C exhibits three thermodynamically stable phases, each with multiple polymorphs: WC (hexagonal and cubic), W_2_C (orthorhombic, trigonal, and hexagonal), and WC_1–*x*_ (cubic).^23,24^ Hexagonal WC space group *P*6̅*m*2 (δ-WC) is considered to be the thermodynamically favored bulk phase.^25^ In contrast, orthorhombic W_2_C space group *Pbcn* (β-W_2_C), trigonal W_2_C space group *P*3̅*m*1 (α-W_2_C), trigonal W_2_C space group *P*3̅1*m* and hexagonal W_2_C (γ-W_2_C) space group *P*6_3_/*mm*3 are metastable phases. Below the eutectoid temperature of 1250 °C, metastable phases other than the most thermodynamically stable δ-WC can be kinetically trapped by controlling the carburization conditions.^26^ Synthesis of phase-pure W_2_C using gaseous carbon precursors remains an elusive goal, with the highest experimentally reported purity reaching ∼92% W_2_C.^25^

The structure of the tungsten oxide (WO_*x*_) precursor plays an important role in governing surface reactivity, the activation of gaseous species, and the diffusion of key intermediates, including graphitic carbon, thereby dictating the overall carburization pathway. This is because tungsten forms multiple oxide phases with stoichiometric and substoichiometric compositions, characterized by oxygen-to-tungsten molar ratios ranging from 2 to 3.^27,28^ Tungsten trioxide (WO_3_) exhibits complex polymorphism with multiple crystal structures, including monoclinic, triclinic, tetragonal, orthorhombic, cubic, and hexagonal phases.^29–31^ These structural polymorphs occur in both stoichiometric and oxygen-deficient variants of WO_3_. The crystallographic configuration of WO_3_ is sensitive to temperature, due to the limited thermal stability of several polymorphs. With increasing temperature, WO_3_ undergoes sequential phase transitions marked by progressive distortions of WO_3_ octahedral units.^32^ Monoclinic WO_3_ is reported as the thermodynamically favored oxide phase under ambient conditions in both bulk and nanoparticle forms, stable up to approximately 350 °C at ambient pressure. At around 350 °C, sequential phase transitions occur from the monoclinic to the orthorhombic phase, followed by a transition to the tetragonal phase near 800 °C.^33^

The carburization pathway of WO_*x*_ to W_*x*_C is influenced by several factors, including the identity of the support, the type and composition of carburization gases, as well as the temperature and gas flow profiles.^34^ Carburization proceeds through precursor activation, followed by surface reactions that generate metallic W and graphitic carbon, which subsequently react to form W_*x*_C.^35^ Silica supports are hypothesized to stabilize the metastable W_2_C phase, however, some literature suggests that W_2_C formation is driven by a particle size effect from the high surface area provided by silica.^25,36^ Other studies highlight chemical effects arising from the formation of Si–O–W bonds during calcination of tungsten precursors, which affect carburization mechanisms and favor W_2_C stabilization.^37^ In a CH_4_/H_2_ environment, the carburization rate of metallic W is limited either by the surface reaction of methane or by the bulk diffusion of carbon, depending on the partial pressure of CH_4_ and the temperature.^38^ However, the underlying mechanism behind formation of metastable W_*x*_C phases and whether the controlling factor is particle size, support effects, or a combination of both remains a subject of debate.

In this work, we address the challenge of W_*x*_C polymorphism by systematically controlling carburization kinetics to achieve phase-selective synthesis. We develop a synthetic strategy to compare widely studied temperature-programmed carburization (TPC) and isothermal carburization (ISC) methods.^39–41^ Our findings reveal that TPC and ISC carburization pathways diverge because of gas and temperature profiles leading to distinct carburization rates and mechanisms. We highlight the effectiveness of TPC in kinetically trapping metastable, carbon-deficient W_*x*_C phases, whereas ISC tends to form carbon-rich WC. Building on this understanding, we selectively synthesize W_2_C and WC using gaseous carbon precursors (CH_4_/H_2_) by leveraging particle size control with TPC. We demonstrate that CH_4_ activation occurs at low temperatures via a surface lattice oxygen (L_O_)-mediated pathway, with metastable W_2_C formation requiring complete reduction of WO_3_ to metallic W and carburization under milder conditions to achieve better kinetic control.

With the ability to selectively synthesize the W_2_C phase, we use the reverse water-gas shift reaction (RWGS) (CO_2_ + H_2_ ⇄ CO + H_2_O; Δ*H*_298_^°^ = +41.1 kJ/mol) as a probe reaction to evaluate the catalytic performance of β-W_2_C. Our reaction studies establish *ex situ* synthesized β-W_2_C as an active and stable catalyst for RWGS, however, because of the pyrophoric nature of tungsten carbide, the surface of *ex situ* β-W_2_C undergoes passivation and activation via high temperature reduction in hydrogen. The resulting complex surface composed of carbides, oxides, oxycarbides, and metallic W, complicates accurate assessment of the intrinsic RWGS activity of the β-W_2_C phase.^42–44^ To overcome this challenge, we have developed an *in situ* synthesis protocol for the selective formation of β-W_2_C. The *in situ* synthesized β-W_2_C exhibits improved catalytic performance and CO space-time yield (STY) compared to the *ex situ* counterpart, owing to elimination of passivation by carburizing the catalyst directly within the reactor prior to RWGS. Collectively, our findings reveal that *in situ* carburization enables the selective formation of β-W_2_C, which is highly active and selective for RWGS.

## 2 Results and Discussion

### 2.1 Structural Analysis of the Precursor (WO_*x*_)

Supported WO_*x*_ on silica is prepared via incipient wetness impregnation (IWI) of ammonium metatungstate hydrate (AMT) onto SiO_2_, followed by drying at 60 °C for 12 h. Subsequently, calcination is performed at 550 °C for 6 h, with a ramp rate of 2 °C min^–1^. Experimental X-ray diffraction (XRD) patterns (Figures 1a and S1) align closely with the theoretical diffraction pattern of monoclinic WO_3_ (space group *P*2_1_/*n*), supporting phase purity and crystallographic consistency. The monoclinic WO_3_ phase, characterized by lattice parameters *a* = 7.30 Å, *b* = 7.53 Å, *c* = 7.68 Å, and β = 90.54° (Figure 1b), is a thermodynamically stable polymorph of WO_3_ at ambient conditions.^45^ The (002) plane exhibits the highest intensity among the reflections (Figure S1), suggesting the (002) facet is the predominant crystallographic orientation, consistent with other studies.^46^ High-resolution transmission electron microscopy (HR-TEM), presented in Figure S2 and Figure 1c, reveals two distinct lattice fringes intersecting at an angle of ∼43°. Fast Fourier transform (FFT) analysis of these fringes shows interplanar spacings (*d*-spacings) of 2.6 Å and 1.8 Å, corresponding respectively to the (220) and (400) crystallographic planes of monoclinic WO_3_ (space group *P*2_1_/*n*).^45,47^ These *d*-spacing values exhibit strong concordance with theoretical lattice dimensions for the (220) and (400) facets, further verifying the crystallographic structure (see Supporting Information (SI) section 1). The observation of the (220) and (400) diffraction planes, coupled with the absence of the (002) plane, indicates that the electron beam is oriented perpendicular to the (002) plane, as it aligns with the crystallographic *c*-axis (refer to Figure 1b). Overall, the FFT data suggests that the surface of the WO_3_ particles is predominantly defined by the (002) planes, identifying the (001) direction as the preferred growth orientation in the monoclinic WO_3_ crystal structure.

**Figure 1:**
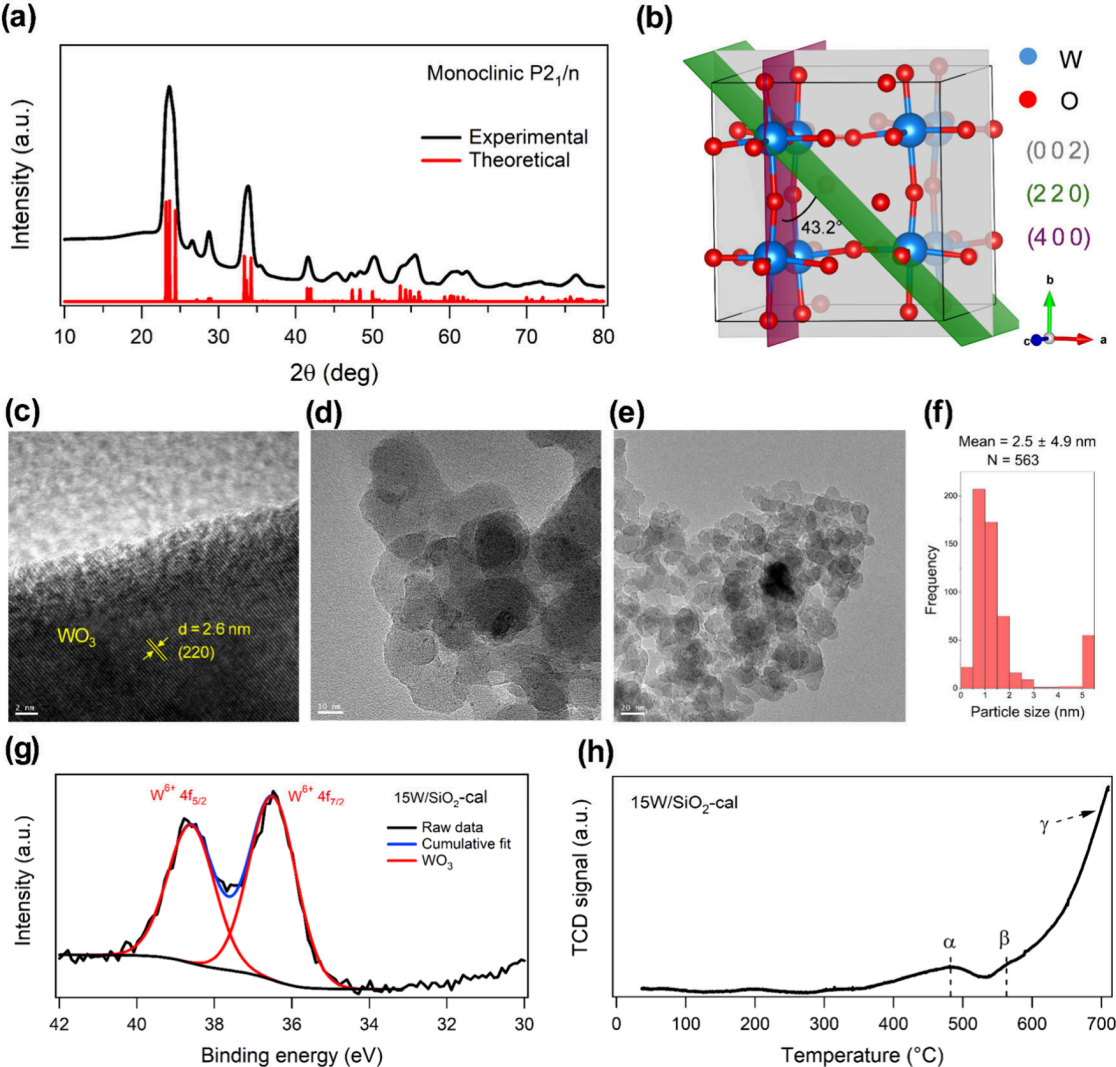
Structural characterization of the calcined 15W/SiO_2_ catalyst. (a) XRD pattern of 15W/SiO_2_-cal, overlaid with theoretical diffraction peaks corresponding to monoclinic WO_3_ (space group *P*2_1_/n). (b) Structural schematic of the monoclinic WO_3_ lattice (space group *P*2_1_/*n*) with emphasis on prominent crystallographic planes (002), (220), and (400). (c) HR-TEM image displaying lattice fringes of WO_3_ from a larger >20 nm particle. (d, e) TEM images of 15W/SiO_2_-cal, revealing uniformly dispersed sub-5 nm WO_3_ particles with occasional agglomeration. (f) Particle size distribution analysis of 15W/SiO_2_-cal via ImageJ software with 10 and 20 nm resolution TEM images. (g) W 4f XPS spectra of 15W/SiO_2_-cal. (h) H_2_-TPR profile of 15W/SiO_2_-cal at a heating rate of 10 °C min^–1^.

TEM images (Figures 1d,e and S3) show that 15W/SiO_2_-cal consists of well-dispersed sub-5 nm WO_3_ particles on the SiO_2_ support, with occasional agglomerates ranging from 5 to 60 nm. Particle size distribution analysis (Figure 1f) indicates a mean particle size of 2.5 nm, with over 90% of particles measured under 5 nm, 8% of particles between 5 and 20 nm, and 2% between 20 and 60 nm. The formation of occasional larger agglomerates can be attributed to the inherent limitations of the IWI method.^6^ Similar to our results, highly dispersed sub-5 nm monoclinic WO_3_ particles on silica with some degree of agglomeration have been previously reported.^48^ Our highly dispersed sub-5 nm particles may create favorable conditions for the selective synthesis of W_2_C, as theoretically predicted size-dependent phase diagrams identify W_2_C as the most thermodynamically stable phase for particle sizes ≤ 10 nm.^36^

Further surface characterization of 15W/SiO_2_-cal by X-ray photoelectron spectroscopy (XPS) in Figure 1g indicates that the surface (sampling depth ∼ 10 nm) consists of WO_3_, as indicated by the W 4f_7/2_ feature at 36.5 eV, along with adventitious carbon (C 1s) at a binding energy (BE) of 284.8 eV. However, the XPS data does not preclude the presence of oxygen vacancies in substoichiometric WO_3–*x*_, where *x* > 0.9. Temperature-programmed reduction under H_2_ (H_2_-TPR) shown in Figure 1h supports a three-step reduction pathway: WO_3_ → WO_2.9_ → WO_2_ → W, with each step corresponding to the α, β, and γ peaks, respectively.^49^ The initial reduction step begins at approximately 340 °C, leading to the formation of hydroxyl groups and oxygen vacancies (V_O_), which we hypothesize have pronounced effects on the carburization mechanism, forming the basis for selective synthesis of W_2_C.

### 2.2 Selective Synthesis of W_2_C

Calcined catalysts are carburized via ISC and TPC, following the profiles detailed in Figure S4. In ISC, the sample is preheated under N_2_ to the desired carburization temperature before switching to the CH_4_/H_2_ gas mixture. In contrast, TPC involves introducing the gas mixture (21% CH_4_/H_2_) at the onset of the process during the gradual 2.1 °C min^–1^ temperature ramp. We exploit this key difference in carburization conditions to control the reduction and carburization kinetics of WO_3_.

We first systematically investigated the effect of carburization conditions on the resultant W_*x*_C phase by performing ISC at four different temperatures (700 °C, 800 °C, 850 °C, and 900 °C) for two durations (2.5 and 5 h). The XRD results in Figure 2a and Figure S5 reveal that ISC lacks the precise control to selectively synthesize carbide phases, with observed concurrent formation of W_2_C and WC. Notably, the temperature profile of 850 °C for 5 h results in a mixture of WC and W_2_C. When the carburization temperature is increased to 900 °C, the γ-WC_1–*x*_ (0.5 < *x* < 1) (200) facet emerges alongside the graphitic carbon (002) facet.^50^ This interesting observation suggests that elevated temperatures drive excessive graphitic carbon deposition on the particle surface, which in turn promotes a sequential phase transformation: W_2_C → WC_1–*x*_ → WC, underscoring the imprecise nature of ISC at controlling the W_*x*_C phase. This observed mechanism is likely because ISC enables the thermodynamically favorable reduction and carburization of WO_3_ when CH_4_ and H_2_ are concurrently introduced at elevated temperatures (>490 °C), resulting in CH_4_ reducing WO_3_ while simultaneously generating graphitic carbon.^51,52^

**Figure 2:**
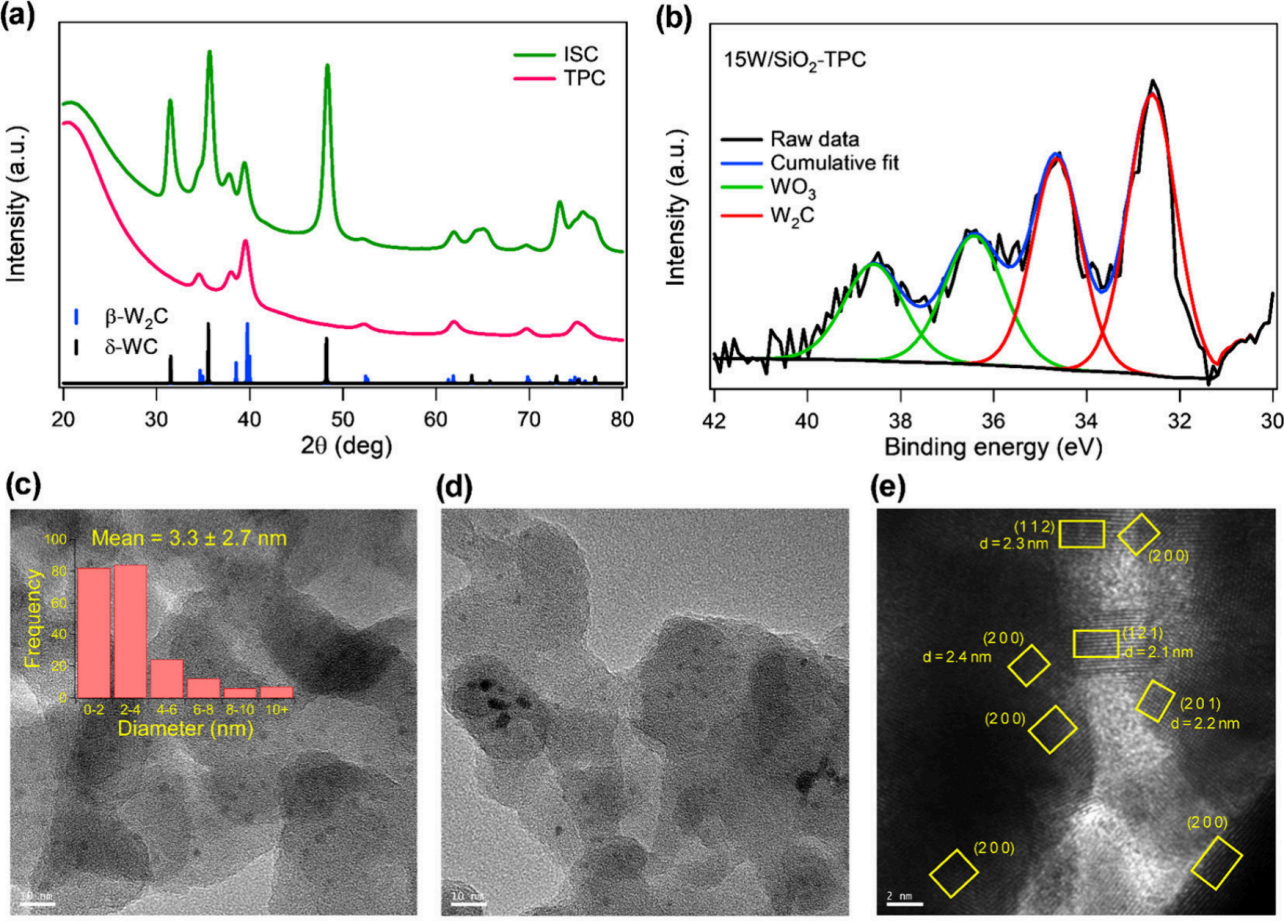
Characterization of carburized 15W/SiO_2_. (a) XRD patterns of carburized 15W/SiO_2_ via ISC and TPC with theoretical diffraction peaks corresponding to β-W_2_C (space group *Pbcn*) and δ-WC (space group *P*6̅*m*2). (b) W 4f XPS spectra of 15W/SiO_2_-TPC-835 °C. (c, d) TEM images of 15W/SiO_2_-TPC-835 °C overlaid with particle size distribution analysis via ImageJ software with 10 and 20 nm resolution TEM images. (e) HR-TEM image of 15W/SiO_2_-TPC-835 °C showing lattice fringes of β-W_2_C.

The ISC pathway promotes methane activation on tungsten oxide (WO_3_) primarily through a L_O_-assisted Mars-van Krevelen (MVK) mechanism.^53^ In this mechanism, CH_4_ reacts with lattice oxygen atoms in WO_3_, facilitating heterolytic C–H bond cleavage. Specifically, a L_O_ atom abstracts a hydrogen atom from CH_4_, forming a hydroxyl (OH*) group on the surface, while the remaining methyl group binds to the surface.^54,55^ Sequential hydrogen abstractions may yield surface carbon from CH_4_. We therefore hypothesize that WO_3_ exhibits a lower activation barrier for the initial C–H bond cleavage via an H-abstraction route, compared to the higher barrier required for homolytic C–H cleavage on metallic W.^56,57^ This lower barrier likely facilitates more extensive carbon deposition during ISC, leading to excess carbon formation and carbon-rich W_*x*_C phases.

In contrast to ISC, TPC favors formation of W_2_C at temperatures slightly above 800 °C. During TPC, the WO_3_ particle surface likely undergoes complete reduction to metallic W prior to the onset of significant carburization. This mechanism arises because reduction of WO_3_ by H_2_ initiates at ∼340 °C (Figure 1h), whereas the dissociation of CH_4_ on metallic W to produce graphitic carbon requires temperatures exceeding ∼490 °C. CH_4_ decomposition on metallic W proceeds via a direct dissociative adsorption mechanism, wherein C–H bonds undergo homolytic cleavage on the metal surface. This mechanism exhibits a relatively high activation energy and requires high temperatures due to the nature of direct C–H bond scission without the aid of lattice oxygen (L_O_).^58^ Gas chromatography (GC) conducted during carburization (Figure S6) supports our hypothesis and reveals CO evolution at ∼490 °C, indicating the onset of carburization. Therefore, the TPC temperature profile likely results in complete reduction of the WO_3_ particle surface to metallic W before significant carburization can occur because the temperature is maintained below 490 °C while remaining above 340 °C for ∼1.25 h (Figure S7).

As evidenced by the XRD results in Figures 2a and S8, β-W_2_C (space group *Pbcn*) is selectively synthesized at a carburization temperature of 835 °C. Rietveld refinement of the XRD data (Figure S9) indicates a phase purity of 99.9% β-W_2_C (see SI section 2 for details of the Rietveld refinement procedure). XPS analysis of W_2_C synthesized at 835 °C via TPC (Figure 2b) reveals that the surface of the catalyst consists of 64% W_2_C and 36% WO_3_. The presence of WO_3_ is attributed to passivation under a diluted O_2_ atmosphere (1% O_2_/N_2_) at 25 °C following carburization, a necessary step to prevent bulk oxidation of pyrophoric tungsten carbide (W_*x*_C).^59–61^ However, the surface oxide layer formed during passivation can lead to misrepresentative characterization of the catalytic activity of W_*x*_C. Surface oxidation of Mo_*x*_C and W_*x*_C has been reported to introduce Bro̷nsted acid sites while reducing Lewis basic sites, thereby diminishing the alkaline activity.^42,62^ In fact, the WO_*x*_ species formed on the carbide surface during passivation introduce acid sites analogous to those present in supported WO_3_.^63^ Consequently, reduction pretreatment is necessary prior to catalytic testing to remove the surface oxide layer. We also observe that the C 1s XPS spectrum (Figure S10) indicates a relative surface W/C atomic ratio of 0.12, suggesting significant surface carbon deposition. Such accumulation of carbon species has been previously reported in the literature and justifies the need for pretreatment in H_2_.^61,64^

TEM imaging (Figure 2c,d) reveals that W_2_C particles are uniformly dispersed across the SiO_2_ support, with occasional larger agglomerates. TEM particle size distribution analysis indicates an average W_2_C particle size of 3.3 nm. This observation is consistent with theoretical predictions of particle size-dependent phase stability in W_*x*_C, which suggests that W_2_C is the thermodynamically favored phase for particle sizes ≤ 10 nm.^36^ HR-TEM (Figure 2e) reveals multiple crystalline domains with well-defined lattice fringes. FFT analysis identifies interplanar lattice spacings of 2.1 Å, 2.2 Å, 2.3 Å, and 2.4 Å, which correspond to the (121), (201), (112), and (200) planes, respectively. Observing these diverse lattice planes suggests the absence of a preferential growth direction, indicating that the W_2_C crystallites exhibit isotropic growth during TPC.

The distinct carburization mechanisms in ISC and TPC highlight the effectiveness of TPC at enabling phase-selective synthesis of W_2_C with controlled particle size, in accordance with theoretical predictions of phase stability for nanoscale tungsten carbides.^36^ However, the underlying mechanism governing the stabilization of the metastable W_2_C phase remains ambiguous. It is unclear whether the stabilization of W_2_C is predominantly controlled by particle-size effects, chemical interactions with the support, or a synergistic combination of both.

### 2.3 Support and Particle Size Effects on W_*x*_C Carburization and Performance

While certain studies in the literature support particle-size effects as the primary driver of W_2_C stabilization, others emphasize the role of support-induced chemical effects. To investigate the support effects on stabilization of the metastable W_2_C phase, WO_*x*_ supported on low and high-surface area alumina (α-Al_2_O_3_ and γ-Al_2_O_3_) are prepared following the same procedure as 15W/SiO_2_-cal, replacing SiO_2_ with either α- or γ-Al_2_O_3_.^25,37^ As an irreducible support, Al_2_O_3_ provides a comparison of the chemical effects of SiO_2_ and Al_2_O_3_ on W_2_C stabilization, while avoiding complications arising from support redox transformations. Additionally, the fumed SiO_2_ (SA = 189.9 m^2^ g^–1^) and γ-Al_2_O_3_ (SA = 129.0 m^2^ g^–1^) used in this study have comparable surface areas, whereas α-Al_2_O_3_ (SA = 3.5 m^2^ g^–1^) has a surface area 2 orders of magnitude lower, enabling us to assess the effect of support surface area on tungsten carburization. Furthermore, a series of WO_3_-supported SiO_2_ samples with varying W loadings (15%, 30%, and 50%) and unsupported WO_3_ are prepared, and all samples are carburized under the same conditions to evaluate particle size and support effects on W_*x*_C synthesis.

As per the XRD results in Figure S11a, the bulk structure of 15W/α-Al_2_O_3_-cal is composed of monoclinic WO_3_ (space group P2_1_ *n*) on trigonal α-Al_2_O_3_ (space group R3̅*C*). Upon carburization, the WO_3_ phase is transformed into β-W_2_C (space group *Pbcn*), as confirmed by the XRD pattern for 15W/α-Al_2_O_3_-TPC-835 °C shown in Figure S11b. On the other hand, the respective catalysts on γ-Al_2_O_3_, 15W/γ-Al_2_O_3_-cal and 15W/γ-Al_2_O_3_-TPC-835 °C, only show XRD peaks corresponding to γ-Al_2_O_3_.^65^ This could be attributed to the highly crystalline nature of γ-Al_2_O_3_ and/or the well-dispersed WO_3_ nanoparticles. Based on TEM particle size distribution analysis (Figures 3a,b and S12), 15W/γ-Al_2_O_3_-TPC-835 °C exhibits the smallest average particle size (2.4 ± 1.3 nm) among the carburized samples, while 15W/α-Al_2_O_3_-TPC-835 °C and 15W/SiO_2_-TPC-835 °C show average particle sizes of 2.6 ± 3.4 nm and 3.3 ± 2.7 nm, respectively.

**Figure 3:**
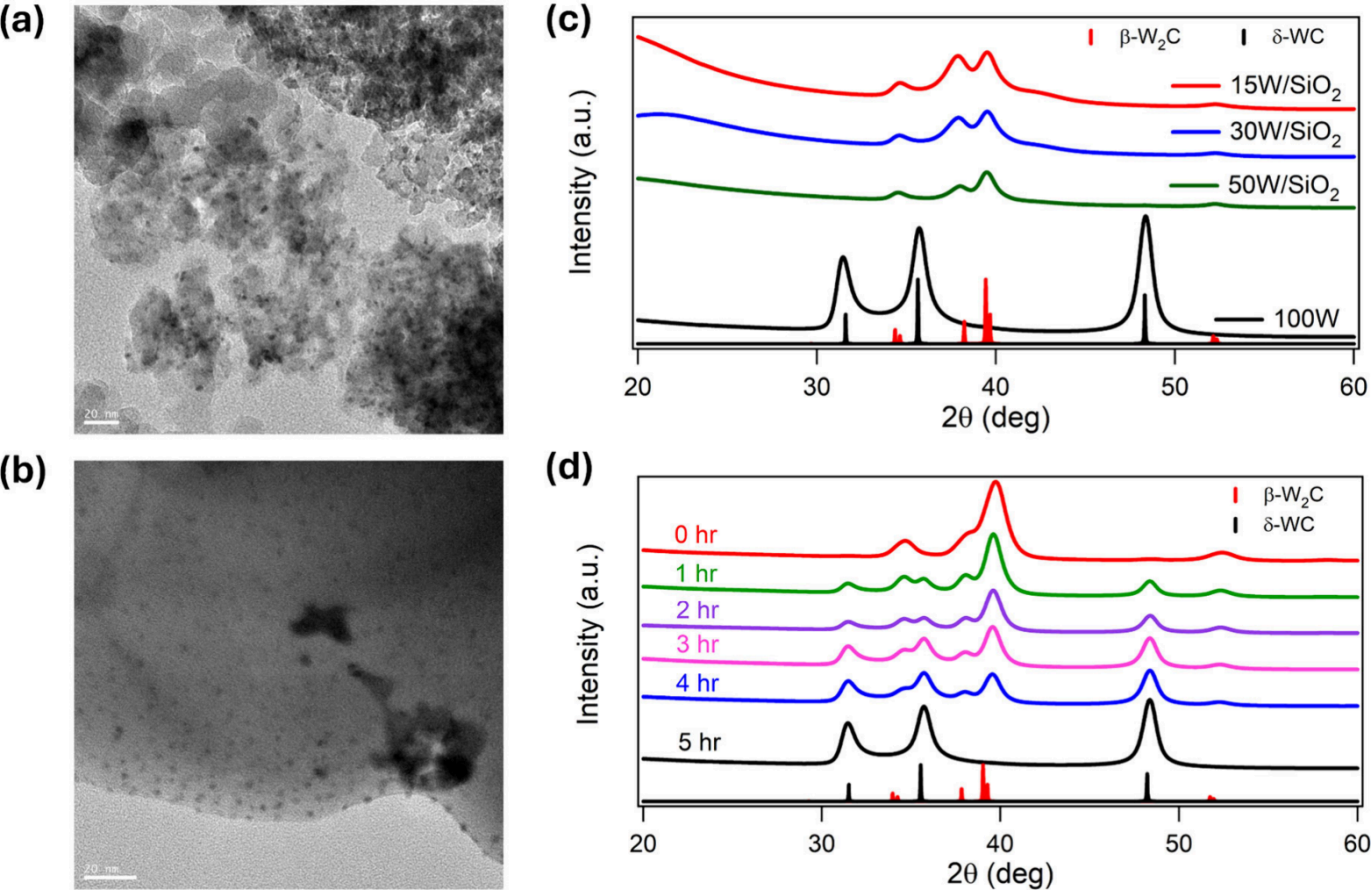
Characterization of support and particle size effects. TEM images of carburized catalysts: (a) 15W/γ-Al_2_O_3_-TPC-835 °C and (b) 15W/α-Al_2_O_3_-TPC-835 °C. (c) XRD patterns of carburized W_*x*_C/SiO_2_ at 835 °C via TPC with varying W weight loadings. (d) XRD patterns showing phase evolution of unsupported W_*x*_C at 835 °C via TPC as a function of carburization extent during quasi *in situ* analysis.

The active surface area of the W_2_C-based catalysts is correlated with the degree of surface oxidation during passivation. Surface oxidation is quantified by measuring the TCD signal area during the first 30 min of exposure to diluted O_2_ (1% O_2_/N_2_) at 35 °C and 1 atm, conditions under which bulk oxidation is negligible. The TCD signal areas (Figure S13), which are correlated with oxygen consumption amounts, for W_2_C supported on SiO_2_, γ-Al_2_O_3_, and α-Al_2_O_3_ are 8.4 × 10^–4^, 11.5 × 10^–4^, and 11.8 × 10^–4^, respectively. These results are within the same order of magnitude and together with TEM particle-size analysis, indicate comparable W_*x*_C surface areas across the three supports.

The catalytic performance of the three carburized catalysts for RWGS is systematically evaluated by temperature-programmed reactions (TPRs) (200–300 °C, 0.25 °C min^–1^, 300 psig, H_2_:CO_2_ = 3:1, GHSV = 135,000 mL h^–1^ g^–1^), revealing comparable catalytic performance (Figure S14). The similar catalytic performance, particle sizes, and W_*x*_C surface areas across the three supported samples suggest that 15W/γ-Al_2_O_3_-TPC-835 °C also contains W_2_C, akin with the other two supports. The average particle size of all three carburized catalysts remains below 10 nm, theoretically favoring the formation of W_2_C according to computational predictions which suggest that metastable phases such as W_2_C are stabilized at smaller particle sizes due to their lower surface energy relative to the high bulk energy penalty.^36^

As the W loading is increased from 15% to 50% on SiO_2_, the average particle size gradually increases from 3.3 to 5.4 nm (Figure S15). However, the particle size remains well below the 10 nm threshold, where the W_2_C phase is thermodynamically stable. Consequently, W loadings of 15%, 30%, and 50% consistently yield β-W_2_C selectively in the bulk structure (Figure 3c). In contrast, the TEM images in Figure S16 show that carburization of unsupported tungsten results in much larger particle sizes with a mean particle size of 96 ± 34 nm, thus leading to the formation of WC, supporting our hypothesis that phase-selective stabilization is predominantly driven by particle size, as we do not observe any differences in phase as a function of the identity of the support.

To further support this hypothesis, particle size-controlled *unsupported* W_*x*_C is synthesized by impregnating AMT onto activated carbon, followed by carbon removal through calcination and subsequent carburization via TPC at 835 °C for 5 h, conditions where unsupported WO_3_ fully converts to WC. This approach yields W_*x*_C with a broad distribution of particle sizes. Removal of the support and subsequent carburization inevitably leads to particle agglomeration, resulting in a heterogeneous particle size distribution. XRD analysis of the W_*x*_C after support removal reveals a mixed phase of WC and W_2_C, suggesting partial stabilization of W_2_C. Consistent with our mixed-phase observation, TEM images (Figure S17) show a mixture of sub-10 nm particles, intermediate particles (10–50 nm), and larger aggregates (>50 nm), while HRTEM images (Figure S18) indicate that the smaller particles consist of W_2_C, whereas the larger aggregates yield WC. These observations further support our hypothesis that phase stabilization of W_2_C is particle size dependent.

The collective evidence presented above demonstrates that stabilization of the metastable W_2_C phase is predominantly a particle size-dependent phenomenon rather than a result of support-specific chemical interactions. The ability to control the particle size below the 10 nm threshold is necessary to selectively synthesize W_2_C, challenging the notion that W–O–Si chemical interactions in W/SiO_2_ play a dominant role in stabilizing the W_2_C phase.^37^ Because of our observation that the W_*x*_C phase is dependent on particle size, the phase evolution of W_*x*_C is inherently linked to the extent of carburization. Specifically, a carbon-deficient W_2_C phase should form prior to the emergence of carbon-rich WC, following the sequential carburization pathway of metallic W → W_2_C → WC_1–*x*_ → WC.

To test our hypothesis on the carburization pathway, we used a quasi-*in situ* XRD experiment where the carburization process is halted at regular time intervals (Figure S19), followed by passive cooling to room temperature and subsequent passivation under 1% O_2_/N_2_. The XRD results with quantitative Rietveld refinement (Figures 3d and S20 and Table S1) suggest a sequential phase transformation from W_2_C to WC as the carburization time, and in turn, the extent of carburization increases. The phase composition of W_2_C decreases from 99.7% to 0%, while that of WC increases from 0.27% to 100% as the carburization time is extended from 0 to 5 h at 835 °C. The direct correlation between the W_*x*_C phase and carburization time supports our hypotheses that (1) tungsten carbide synthesis is governed by sequential phase transformations; and (2) stabilization of W_2_C is predominantly governed by particle size.

### 2.4 Investigations of the WO_*x*_ Carburization Mechanism

To better understand the kinetics of W_*x*_C formation via the sequence of W_2_C → WC_1–*x*_ → WC and support our above findings regarding W_2_C stabilization, we conducted detailed mechanistic studies during *in situ* TPC of WO_3_/SiO_2_ using diffuse reflectance infrared Fourier transform spectroscopy (DRIFTS). These experiments provide insight into the rate of methane consumption, surface intermediates, and product evolution during the early stages of carburization, revealing key activation steps in the CH_4_/H_2_ environment.

To minimize errors arising from signal attenuation due to increased optical opacity as the sample darkens during carburization, only the initial stages of the mechanism are investigated. The maximum temperature is restricted to 700 °C due to instrument limitations, and the ramp rate is 10 °C/min to shorten the carburization duration and minimize signal attenuation. A signal attenuation factor (η) is defined in eq 1, under the assumption that the rate of signal attenuation is negligible during the initial phase of carburization. As a result, the gas-phase CH_4_ concentration (*C*_CH_4_,g_) exhibits a linear correlation with the area of the CH_4_ symmetric stretching vibrational mode (ν_3_) at ∼3016 cm^–1^ (*A*_CH_4__) (Figures 4a and S21), as described in eq 1, providing a basis for interpreting the CH_4_ consumption rate (*R*_CH_4__). Under the assumption that the volumetric flow rate of the synthesis gas mixture (*v*_O_) is constant under dilute conditions, *R*_CH_4__ simplifies to eq 2. For IR peak assignments and comparisons with literature values, refer to Figure 4b and Table S2.

$$C_{{\mathrm{CH}}_{4},g}=\alpha \left(A_{{\mathrm{CH}}_{4}}+\eta \right)$$ 1

$$R_{{\mathrm{CH}}_{4}}=v_{O}\cdot \alpha \cdot \Delta \left|A_{{CH}_{4}}\right|$$ 2

In eqs 1 and 2, α represents the extinction coefficient of CH_4_, and |*A*_CH_4__| denotes the peak area magnitude of the CH_4_ asymmetric stretch (ν_3_) relative to the background (Figure S21). As shown in Figure 4c, CH_4_ undergoes slow dissociation on WO_3_ under low temperature below 490 °C, as evidenced by low concentrations of gas-phase species that are not detected by GC (Figure S6), and the carburization rate progressively increases as the temperature is increased to 320 °C. Throughout the 25–320 °C temperature range, CO is the predominant product, with a concurrent increase in both gas-phase CO and chemisorbed CO* (Figure S22). This observation is notable, considering the symmetric structure and low polarizability of CH_4_, which results in a high dissociation energy for the first C–H bond (∼439 kJ mol^–1^ at 25 °C), making CH_4_ activation at low temperatures particularly challenging.^66^ The possibility of this phenomenon being an experimental artifact due to preadsorbed species can be excluded, as the samples undergo thermal treatment under Ar at 400 °C for 2 h to remove any preadsorbed contaminants.

**Figure 4:**
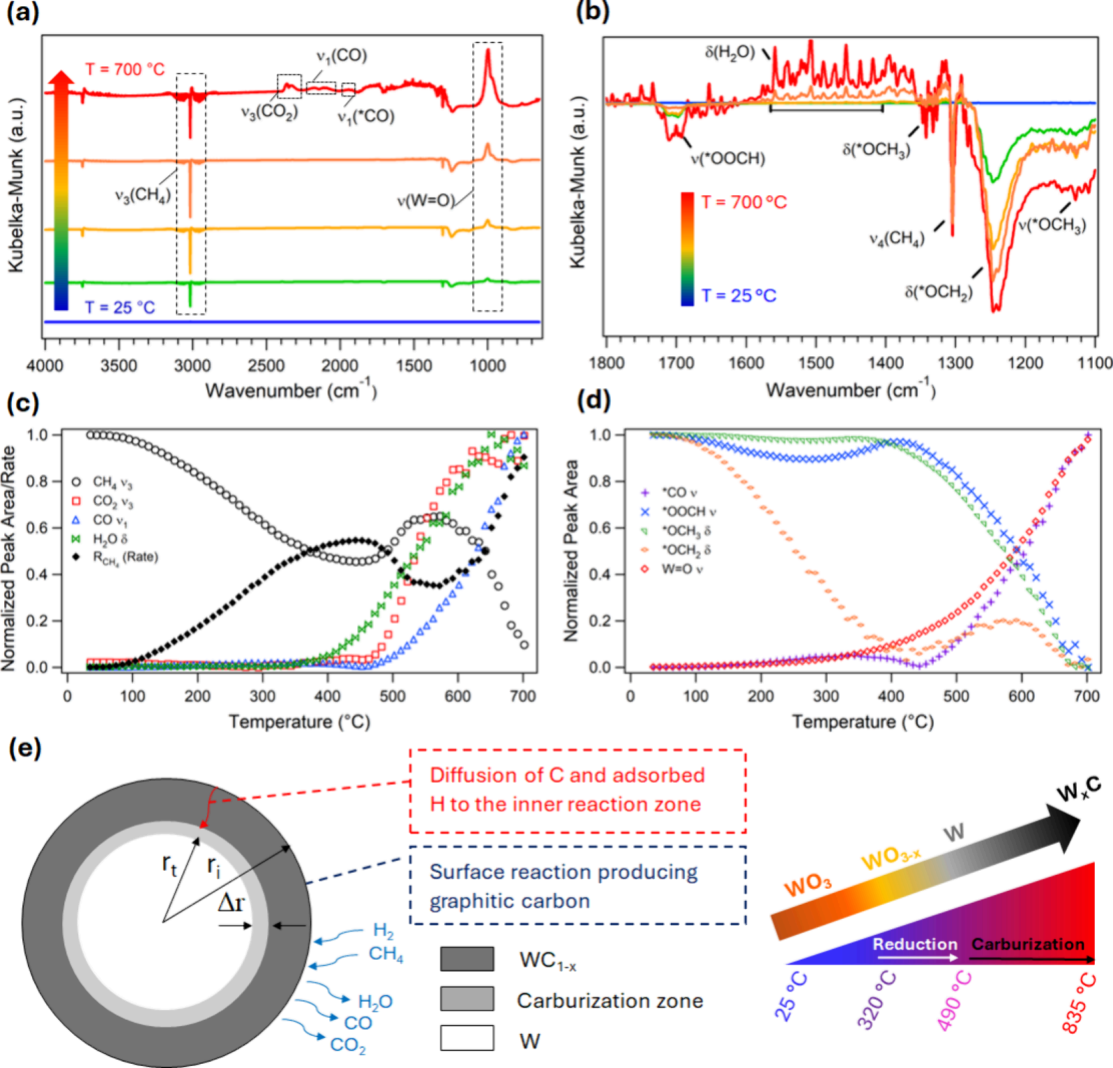
Mechanistic analysis of supported WO_3_ (15W/SiO_2_-cal) carburization via temperature-programmed carburization (TPC). (a) Time-resolved DRIFTS spectra recorded during *in situ* TPC of 15W/SiO_2_-cal. (b) Zoomed-in DRIFTS spectra, highlighting the surface intermediates identified during carburization. (c) Evolution of reactants (CH_4_) and products (CO, CO_2_, and H_2_O) during carburization, showing the normalized peak areas of the respective IR bands as a function of temperature. Also plotted is normalized rate of CH_4_ consumption (*R*_CH_4__). (d) Normalized peak area of surface species during carburization as a function of temperature. (e) Schematic of the proposed carburization mechanism of W_2_C via temperature-programmed carburization (TPC) with particle radius, *r*_i_ and radial distance to the reaction front *r*_*t*_ at time *t*. Reduction of WO_3_ initiates at ∼320 °C, forming metallic W prior to the onset of carburization at ∼490 °C (TPC ramp rate: ∼2 °C min^–1^).

Low-temperature CH_4_ activation has been previously reported for metal oxides such as IrO_2_ (110) and single-atom doped TiO_2_ (110), where specific surface L_O_ sites play an important role in facilitating CH_4_ activation.^54,67^ The observed low-temperature CH_4_ activation on WO_3_ is likely attributed to the presence of L_O_ sites on the high-surface-energy (002) facets of WO_3_, which dominate the surface of 15W/SiO_2_-cal, as shown in Figure 1.^68,69^ Therefore, it is hypothesized that the surface L_O_ of WO_3_ (002) facilitate C–H bond activation and subsequent oxidation to CO through a MVK mechanism. Typically, in a MVK mechanism, CH_4_ activation on a metal oxide proceeds via a four-center transition state, where the carbon atom binds to the metal center while hydrogen associates with a lattice oxygen atom. However, for CO formation to occur, the carbon interacts directly with the lattice oxygen site. As shown in Figure S23, under low-temperature conditions (<320 °C), the emergence of a H_*y*_WO_*x*_ phase is observed, with the formation rate increasing with temperature. This observation supports our hypothesis that hydrogen migrates into the WO_3_ bulk lattice to form the H_*y*_WO_*x*_ phase, thereby making lattice oxygen available for CO formation. The CH_4_ consumption rate begins to decrease as the temperature exceeds 320 °C, which corresponds to the onset of WO_3_ reduction by H_2_. This shift in the carburization mechanism is evidenced by an increase in H_2_O_(g)_, consistent with H_2_-TPR (Figure 4c, Figure 1g). We hypothesize that the reduction of WO_3_ by H_2_ depletes the concentration of L_O_, leading to the observed decline in *R*_CH_4__.

The slow reduction of WO_3_ by CH_4_ under mild temperature conditions, followed by an accelerated reduction by H_2_ above 320 °C, is further corroborated by the appearance of an emerging IR band at 850–1120 cm^–1^, which is attributed to terminal W = O stretching vibrations.^70^ As WO_3_ undergoes reduction, forming partially reduced and hydrated WO_*x*_ species, a broad IR peak with multiple shoulders emerges within this range, indicating a progressive increase in the degree of surface reduction.^71,72^ The broadening and multiplicity of this peak suggest the formation of various H_*y*_WO_*x*_ substoichiometries, which serve as indicators of incomplete reduction of WO_3_.

As expected, the concentrations of CO and *CO also decrease with the decreasing rate of methane consumption (Figure 4c,d). The observed inverse relationship between CO and the surface concentrations of intermediate species *OOCH, *OCH_3_, and *OCH_2_, suggests a sequential reaction pathway for CH_4_ oxidation to CO following the sequence: *CH_4_ → *OCH_3_ → *OCH_2_ → *OOCH → *CO → CO.^73^ Furthermore, the results indicate increased generation of CO_2_ under reducing conditions, suggesting that in the presence of excess *OH, the intermediate *OOCH can react with *OH to yield CO_2_ and H_2_O (Table S3).

The CH_4_ consumption rate begins to increase rapidly around 490 °C, a temperature sufficient to enable CH_4_ dissociation in the absence of lattice oxygen, suggesting a shift from an L_O_-mediated C–H bond heterolysis pathway to a homolysis mechanism.^56^ Consequently, the availability of graphitic carbon increases at elevated temperatures and facilitates carburization of WO_*x*_ to W_*x*_C. Based on these findings, we propose a single-particle carburization model for the TPC method, as detailed in SI section 3, which incorporates sequential reduction and carburization of WO_3_ into W_*x*_C. The model delineates three distinct reaction regimes (*T* < 320 °C, 320 < *T* < 490 °C, and *T* > 490 °C), each characterized by specific elementary steps (Table S3). Reduction and carburization initiate at the particle surface and advance inward toward the core. The initial rate of carburization is governed by the rate-limiting surface reaction that generates graphitic carbon, which subsequently diffuses into the solid matrix to facilitate phase transformation from metallic W to W_*x*_C (Figure 4e).

### 2.5 Catalytic Performance of *Ex Situ* Synthesized W_2_C

Based on our characterization data, the 15W/SiO_2_-TPC-835 °C discussed in the bulk of this study that is synthesized *ex situ* contains well-dispersed β-W_2_C in the bulk phase. The catalytic performance for the RWGS reaction is evaluated with temperature-programmed reactions (200–300 °C). Additionally, a 100 h time-on-stream (TOS) test at 300 °C is conducted for 15W/SiO_2_-TPC-835 °C to assess the long-term stability and potential structural transformation of the metastable phase (300 °C, 300 psig, H_2_:CO_2_ = 3:1, GHSV = 135,000 mL h^–1^ g^–1^). In both cases, the catalysts are reduced in H_2_ at 350 °C for 2 h prior to reaction to ensure reduction of the passivation layer.

The results of the TPR-200-300 °C (Figure 5a) indicate β-W_2_C is an active catalyst, with the CO_2_ conversion reaching ∼26% at 300 °C. As the temperature is ramped from 200 to 300 °C, the CO_2_ conversion gradually increases and CO selectivity decreases, while the selectivity toward C_2_ (ethane and ethylene) and C_3+_ hydrocarbons increases, with a notable shift occurring in the conversion and selectivity above 260 °C. This observed shift in product selectivity suggests a temperature-dependent transformation of the reaction pathway, potentially favoring chain growth mechanisms and hydrocarbon formation at higher temperatures. This shift in catalytic performance is evidenced by the total selectivity toward C_2_ and C_3+_ hydrocarbons, which reaches 22% at 300 °C.

**Figure 5:**
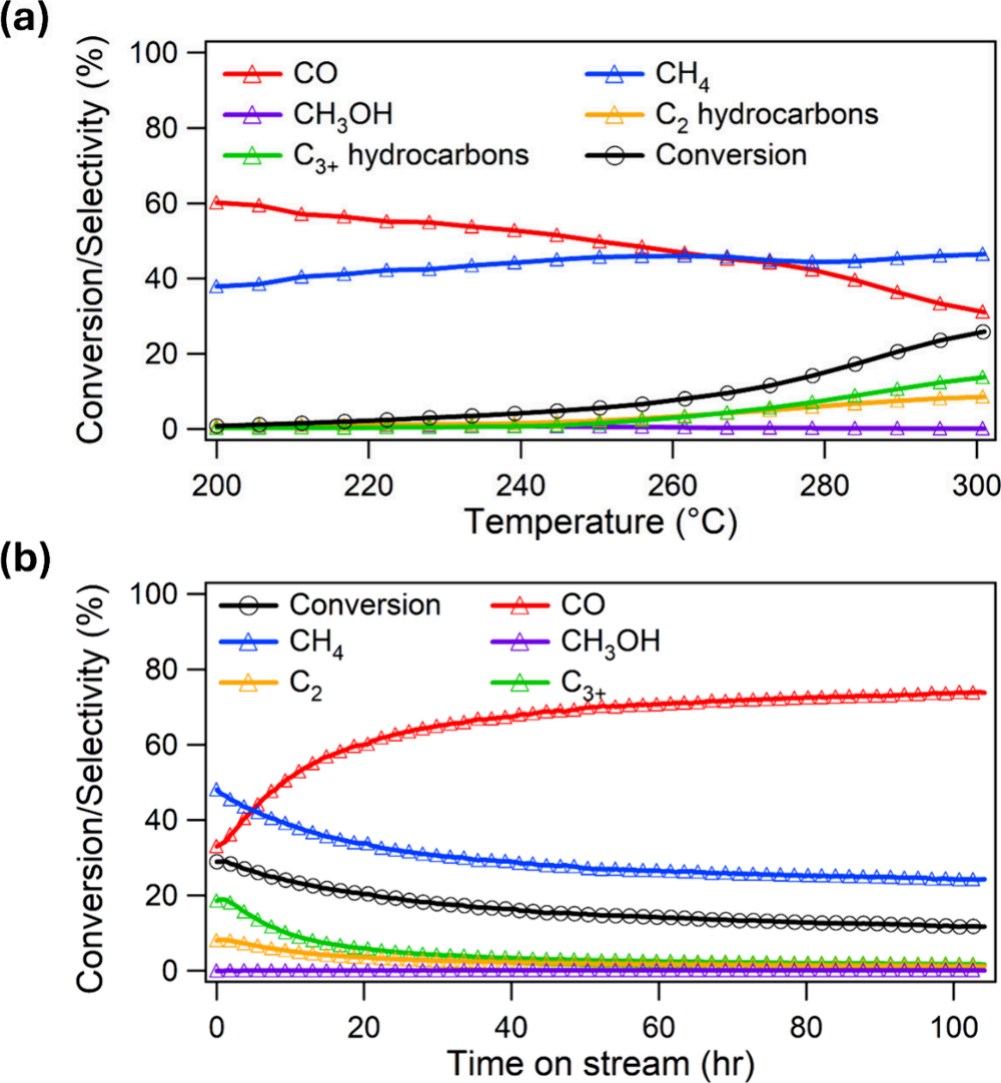
Catalytic performance of *ex situ* W_2_C/SiO_2_ for CO_2_ hydrogenation. (a) CO_2_ conversion and product selectivity during TPR-200-300 °C over 15W/SiO_2_-TPC-835 °C. (b) TOS CO_2_ hydrogenation performance of 15W/SiO_2_-TPC-835 °C at 300 °C. Reaction conditions: H_2_:CO_2_ = 3:1, *P* = 300 psig, GHSV = 135,000 mL h^–1^ g^–1^. All samples were prereduced at 350 °C for 2 h prior to reaction. The carbon balance for all reactions is within ±1% of 100%.

The isothermal TOS study of 15W/SiO_2_-TPC-835 °C (Figure 5b) reveals a gradual decline in CO_2_ hydrogenation activity over the first 50 h of operation. Concurrently, a notable increase in CO selectivity is observed, accompanied by a decrease in CH_4_, C_2_, and C_3+_ hydrocarbon selectivity. The initial induction period of ∼50 h can likely be attributed to the presence of metallic W formed during the reduction pretreatment, which gradually carburizes in the presence of deposited carbon. We observe carbon deposition on the catalyst surface during carburization via XPS in Figure S10. The crystallite size of W_2_C determined via Scherrer analysis from XRD is ∼2.5 nm for the post-TOS sample, in contrast to ∼12.6 nm for the as-synthesized sample (see SI section 4 for details). This reduction in crystallite size likely indicates partial restructuring under reaction conditions, likely due to the formation of defects or amorphous surface layers, and/or the segmentation of larger crystallites into smaller coherently diffracting domains. After 50 h, the catalytic performance reaches steady-state, suggesting that the catalyst structure equilibrates, yielding consistent activity (∼14% conversion) and selectivity (∼78% CO) for the remainder of the TOS experiment. The post-TOS XRD analysis (Figures S24 and S25) confirms the retention of W_2_C with 99.86%, indicating the stability of the bulk phase of the catalyst under reaction conditions.

### 2.6 *In Situ* Synthesis and Performance of W_2_C

The passivation step required for *ex situ* synthesis inevitably generates a surface oxide layer that must be removed to eliminate artifacts, which could obscure the intrinsic catalytic behavior and lead to inaccurate performance evaluation. However, such reductive pretreatments typically produce a complex surface comprising a heterogeneous mixture of oxides, carbides, oxycarbides, and metallic tungsten.^19,42,43^ This compositional heterogeneity complicates precise surface characterization under reaction conditions, introducing substantial uncertainty regarding the kinetically relevant surface structure of the catalyst. To circumvent these confounding factors and better understand the intrinsic activity of β-W_2_C, we employ an *in situ* synthesis strategy that obviates the need for passivation and thus prevents surface oxidation prior to evaluating the catalytic performance.

In this approach, a packed bed of supported WO_3_/SiO_2_ is directly carburized by *in situ* ISC and TPC methods under a 21 vol % CH_4_/H_2_ mixture. The *ex situ* carburization conducted in the tube furnace differs fundamentally from the *in situ* process owing to variations in heat and mass transfer, and measured temperatures that differ by >100 °C, although in both cases the actual temperature of the sample is similar (Figure S26).^74,75^ The temperature measurement discrepancy between *in situ* and *ex situ* carburization is because temperature within the reactor is measured via a thermocouple in contact with the catalyst bed, whereas temperature in the tube furnace, used for carburizing *ex situ* samples, is in contact with the exterior of the quartz tube.^76^

Determining the conditions required to synthesize W_2_C is challenging because we could not directly characterize the crystal structure of the catalyst prior to reaction. Similar to the *ex situ* samples, we quickly determined that *in situ* ISC exhibits poor control over the W_x_C phase (Figure S27). In our initial attempt at replicating the TPC synthesis with *in situ* carburization at 835 °C, postreaction XRD analysis (Figure S28) revealed that the sample predominantly consists of W_2_C with minor amounts of WC_1–*x*_ where 0.5 < *x* < 1, indicating overcarburization. The formation of WC_1–*x*_ during the TPR-200-300 °C is unlikely, as the reaction temperatures are insufficient to drive further carburization as evidenced from our mechanistic study. We then decreased the *in situ* carburization temperature to 713 °C, which primarily yielded W_2_C with traces of metallic W. Reduction of W_2_C during the reaction is also unlikely given the reaction temperatures, and postreaction XRD analysis of the *ex situ* catalyst (Figure S24 and S25).

To further tune the structure and performance of W_*x*_C, we used CO STY during CO_2_ hydrogenation as a surrogate for structure, complemented by postreaction XRD to determine the resulting W_*x*_C phase. As demonstrated in Figure 6a, *in situ* carburization via TPC at 713 °C for 2 h significantly increases CO STY compared to an equivalent *ex situ* carburization profile at 835 °C. We arrived at these carburization parameters by using CO STY as the objective function, while varying the residence time, which is inversely proportional to the superficial velocity (controlled by adjusting the feed-gas flow rate). As shown in postreaction XRD analysis in Figure S29, shorter synthesis gas residence times lead to higher catalytic activity. Catalysts carburized under longer residence times predominantly consist of W_2_C with minor amounts of WC, indicative of overcarburization resulting from prolonged exposure to the carburizing gas. In contrast, samples carburized under shorter residence times primarily exhibit W_2_C with traces of metallic W within the bulk crystal structure. The catalytic activity is further optimized by varying the carburization duration. As shown in the catalytic performance and XRD data in Figures 6 and S30, the highest CO STY is achieved after 2 h of carburization, which corresponds to W_2_C with minor amounts of metallic W. The shortest carburization time (1 h) yields the highest intensity of metallic W reflections, indicating incomplete carburization to W_2_C. In contrast, extending carburization to 5 h causes CO STY to decrease as a result of overcarburization of the surface. Collectively, our observations indicate that a 2 h carburization duration provides the optimal balance between W_2_C formation and suppression of WC_1–*x*_ (0.5 < *x* ≤ 1), resulting in the highest catalytic performance and supporting our hypothesis that W_2_C is the most catalytically relevant phase for CO_2_ hydrogenation.

**Figure 6:**
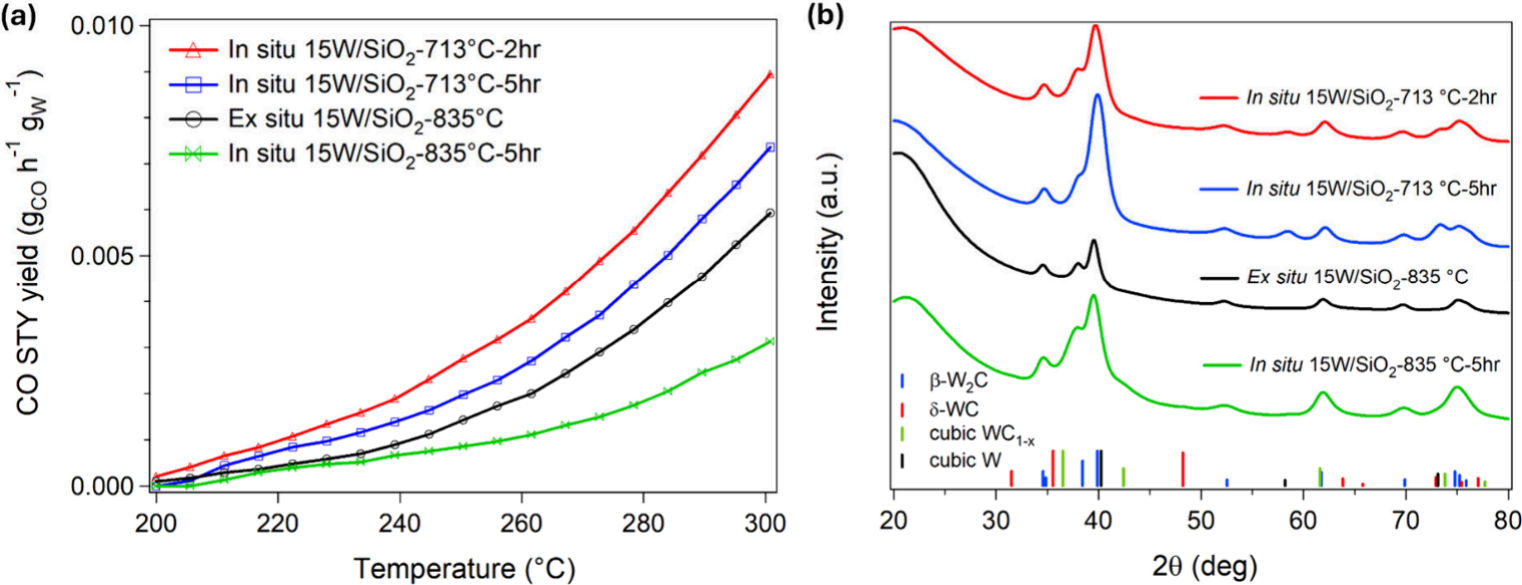
Comparison of RWGS performance and structure of *in situ* vs *ex situ* W_*x*_C. (a) CO STY during RWGS from 200 to 300 °C, H_2_:CO_2_ = 3:1, *P* = 300 psig, GHSV = 27,000 mL h^–1^ g^–1^. The *ex situ* synthesized catalyst was prereduced at 350 °C for 2 h prior to reaction. The carbon balance for all reactions is within ± 1% of 100%. (b) Postreaction XRD patterns of 15W/SiO_2_-*T* °C-*t* hr-TPC, where *T* and *t* indicate carburization temperature and duration, respectively.

## 3 Conclusions

Tackling the challenges associated with multiphase behavior and complex polymorphism in transition metal carbides is not only fundamentally important, but also industrially relevant for developing earth-abundant, active catalysts. This study addresses the challenge of W_*x*_C polymorphism by demonstrating a framework for phase-selective synthesis via controlling carburization kinetics. By maintaining particle sizes below 10 nm, β-W_2_C is preferentially stabilized under TPC conditions at 835 °C. Our findings establish that stabilization of metastable W_2_C is primarily dictated by particle size rather than support-specific interactions. However, the limited control over the surface composition of *ex situ* synthesized W_2_C, arising from the required postsynthesis passivation, leads to a complex surface structure comprising carbides, oxides, oxycarbides, and metallic W. To mitigate these confounding variables, we demonstrate *in situ* synthesized β-W_2_C exhibits higher catalytic performance for RWGS compared to its *ex situ* counterpart. By addressing W_*x*_C multiphase behavior, the insights gained from this study extend beyond W_*x*_C, providing a broader framework for tuning phases, and in turn, performance in transition metal carbide systems across diverse applications.

## 4 Methods

### 4.1 Catalyst Preparation

Supported WO_*x*_ catalysts are synthesized via incipient wetness impregnation (IWI) of ammonium metatungstate hydrate (AMT) (99.99% trace metals basis, Alfa Aesar) onto the respective supports, followed by calcination in air. To prepare WO_*x*_ supported on SiO_2_ with a target loading of 15 wt % W (15W/SiO_2_-cal), AMT is impregnated onto Silicon­(IV) oxide (SiO_2_), amorphous fumed (surface area: 175–225 m^2^ g^–1^, Thermo Scientific) via IWI. The impregnated sample is then dried at 60 °C for 12 h and subsequently calcined in air at 550 °C for 6 h in a box furnace (Lindberg Blue M), with a heating ramp rate of 2 °C min^–1^. Other supported catalysts are synthesized using the same procedure with different supports, including γ-Al_2_O_3_ (γ-phase aluminum oxide) (surface area: 100–150 m^2^ g^–1^, 99.9% metal basis, Alfa Aesar) and α-Al_2_O_3_ (α-phase aluminum oxide) (surface area: 2–4 m^2^ g^–1^, 99.9% metal basis, Alfa Aesar).

Calcined catalysts are carburized in a horizontal tube furnace using two methods: isothermal carburization (ISC) and temperature-programmed carburization (TPC). In the TPC method, a reactive gas mixture consisting of 21% CH_4_/H_2_ is introduced at a flow rate of 155 mL min^–1^ with a gas stream velocity of 10 cm s^–1^. The sample is exposed to this gas mixture at 25 °C for 30 min to allow for equilibration before ramping to the carburization temperature (the gas and temperature profile are provided in Figure S4). Following a 5-h carburization period, the gas flow is switched from 21% CH_4_/H_2_ to pure H_2_ and maintained for an additional 0.5 h to remove excess carbon. The sample is then passively cooled to 25 °C under a flow of H_2_. Upon reaching 25 °C, the catalyst is subjected to passivation in 1% O_2_/N_2_ at 25 °C for 3 h to prevent uncontrolled oxidation upon exposure to air.

In the ISC method, N_2_ is introduced at a flow rate of 155 mL min^–1^ with a gas stream velocity of 10 cm s^–1^ and allowed to equilibrate at 25 °C for 30 min. The temperature is then ramped to the carburization temperature under N_2_ (the temperature profile is also provided in Figure S4). Once the carburization temperature is reached, the gas flow is switched from N_2_ to 21% CH_4_/H_2_. When the carburization duration is completed, the flow is switched to H_2_ and maintained for an additional 0.5 h to remove excess carbon. The sample is subsequently passively cooled to 25 °C under a flow of H_2_. Upon reaching 25 °C, the catalyst is subjected to passivation in 1% O_2_/N_2_ at 25 °C for 3 h to prevent uncontrolled oxidation upon exposure to air.

Unsupported WO_3_ is synthesized by direct calcination AMT in air at 550 °C for 6 h in a box furnace (Lindberg Blue M), using a heating ramp rate of 2 °C min^–1^. To obtain unsupported W_*x*_C, the calcined WO_3_ samples are carburized via temperature-programmed carburization (TPC) at 835 °C, following similar conditions as the supported catalysts (Figure S4). Based on insights from quasi-*in situ* XRD analysis, the carburization duration is adjusted to achieve phase-selective synthesis, with 0 h for W_2_C and 5 h for WC. To obtain metallic W, unsupported WO_3_ is reduced under a pure H_2_ flow (122 mL min^–1^) using the same temperature profile employed for WC synthesis. All samples are subsequently passivated following the procedure described above.

To synthesize support-removed W_*x*_C, AMT is impregnated onto activated carbon, dried, and calcined following the procedure described above. During calcination at 550 °C for 6 h, the activated carbon is fully combusted, yielding support-removed WO_*x*_, which is subsequently carburized via TPC at 835 °C for 5 h, following the method outlined for synthesizing unsupported WC above.

### 4.2 X-ray Diffraction (XRD)

XRD measurements are conducted using a Rigaku XtaLAB Synergy-S diffractometer equipped with a HyPix-6000HE hybrid HPC detector. Powder samples are prepared by mounting them on a 0.1 mm nylon loop coated with a thin layer of viscous oil. Cu Kα radiation (λ = 1.54184 Å) is employed, generated by a PhotonJet-S microfocus source operating at 50 kV and 1 mA. The data are collected at room temperature (293 K) with a sample-to-detector distance of 34 mm. Two sequential combined ω–ϕ “Gandolfi” scans are performed, each with an acquisition time of 300 s. In the first scan, ω is varied from −62.00° to 31.00°, while ϕ is continuously rotated through 720°, with θ fixed at −42.127° and κ at 70.000°. The second scan involved ω being adjusted from −31.00° to 61.00°, with ϕ again rotated through 720°, and fixed angles of θ = 40.877° and κ = −70.000°.

### 4.3 Transmission Electron Microscopy (TEM)

TEM analyses are conducted using a FEI Tecnai G2-F20 scanning transmission electron microscope (STEM) operated at an accelerating voltage of 200 kV. Dry powder samples are directly deposited onto carbon-coated copper grids without any pretreatment prior to transferring the grids into the TEM chamber. Particle size distributions and fast Fourier transform (FFT) analyses are performed using ImageJ software.

### 4.4 X-ray Photoelectron Spectroscopy (XPS)

XPS analyses are conducted using a Kratos Axis Ultra DLD spectrometer equipped with a monochromatic Al Kα X-ray source (1486.6 eV). The X-ray source is operated at 200 W under ultrahigh vacuum (UHV) conditions of 3.0 × 10^–8^ mbar. Spectra are collected in slot aperture analyzer mode, corresponding to an analyzed area of approximately 300 μm × 700 μm. The electron collection angle (θ) is maintained at 0°. Survey scans are acquired over a binding energy range of 0–1200 eV using a step size of 1.0 eV, a dwell time of 200 ms, and a pass energy of 120 eV. For high-resolution core-level spectra, a step size of 0.1 eV and a pass energy of 20 eV are employed, with data averaged over ten scans to enhance the signal-to-noise ratio. The XPS data are processed using CasaXPS software, and spectra are fitted using Shirley backgrounds and Gaussian–Lorentzian functions. The adventitious C 1s peak is set to 284.8 eV as a reference, and a uniform binding energy shift is applied to all spectra to correct for potential charging effects.

### 4.5 Temperature-Programmed Reduction under H_2_ (H_2_-TPR)

H_2_-TPR experiments are conducted using a Micromeritics AutoChem II analyzer equipped with a thermal conductivity detector (TCD). A 150 mg sample of 15W/SiO_2_-cal sample is weighed and loaded into a sample tube. The sample is initially purged under an argon (Ar) flow of 25 mL min^–1^ at ambient temperature (35 °C) for 2.5 h. Subsequently, a 5% H_2_/Ar mixture is introduced at a flow rate of 50 mL min^–1^ and allowed to equilibrate for 15 min prior to the temperature ramp. The temperature is then increased to 700 °C at a heating rate of 10 °C min^–1^.

### 4.6 Gas Chromatography Analysis of Carburization Products

To analyze the evolution of gaseous products during TPC at 835 °C, the concentrations of products are measured using an in-line Agilent Technologies 7890B gas chromatograph equipped with both a flame ionization detector (FID) and a thermal conductivity detector (TCD). A 100 mg sample of calcined WO_3_/SiO_2_ (15W/SiO_2_-cal) is loaded into a stainless-steel tube (outer diameter: 6.35 mm, inner diameter: 4.57 mm) mounted horizontally in the furnace. The temperature and gas profiles used are identical to those described in Catalyst Preparation, with a total flow rate of 47 mL min^–1^, corresponding to a superficial velocity of 50.2 cm s^–1^. The mole fractions of gas-phase species are calibrated by correlating the peak areas obtained for each compound to their respective concentrations in a calibration gas standard. No significant changes in the concentrations of CH_4_ and H_2_ are detected during carburization, as their consumption rates are below the limit of detection (LOD) of the analytical system. Similarly, CO_2_ is not observed, likely due to its concentration being below the LOD. Furthermore, the GC used in this study cannot quantify H_2_O. As shown in Figure S6, CO is the sole carbon-containing product detected during the carburization process. However, *in situ* diffuse reflectance Fourier transform infrared spectroscopy (DRIFTS) studies reveal that CH_4_ is activated at low temperatures and can be oxidized to CO at a slow rate below 490 °C through the involvement of lattice oxygen. Such low concentrations of CO below 490 °C are inferred to be below the LOD of the GC. A rapid evolution of CO is observed at approximately 490 °C, which is likely indicative of the onset of carburization, consistent with the results obtained from DRIFTS studies.

### 4.7 Quasi-*In Situ* XRD Analysis of TPC for Unsupported WO_3_

Unsupported WO_3_ is synthesized following the procedure outlined in Catalyst Preparation. An ∼1 g sample is loaded into a porcelain crucible and placed in a horizontal tube furnace. The sample underwent temperature-programmed carburization (TPC) at 835 °C under a 21% CH_4_/H_2_ atmosphere (Figure S19). The carburization is halted at different time intervals (0–5 h, in 1-h increments) by switching the gas flow from 21% CH_4_/H_2_ to pure H_2_. The sample is subsequently exposed to pure H_2_ for an additional 0.5 h at 835 °C to remove graphitic carbon from the surface before being passively cooled to room temperature (25 °C) under H_2_. It is then passivated in 1% O_2_/N_2_ for 3 h prior to XRD analysis. A fresh sample is used for each carburization duration to ensure accurate phase analysis.

### 4.8 *In Situ* DRIFTS Investigation of the Carburization Mechanism

To investigate the initial reaction mechanism of supported WO_*x*_ carburization via temperature-programmed carburization (TPC), *in situ* DRIFTS measurements are conducted using a Nicolet iS50 spectrometer (Thermo Scientific) equipped with an MCT/A detector. Approximately 50 mg of 15W/SiO_2_-cal, diluted 1:10 wt % with KBr, is loaded into a Harrick Praying Mantis DRIFTS cell with ZnSe windows. The cell is purged with Ar (10 mL min^–1^) for 30 min, then heated to 400 °C at a rate of 10 °C min^–1^ and degassed at 400 °C for 2 h under an Ar atmosphere. After degassing, the sample is cooled to 25 °C under Ar flow. A diluted synthesis gas mixture consisting of 21% CH_4_/H_2_ (4 mL min^–1^) and Ar (10 mL min^–1^) is then introduced to enhance experimental sensitivity. The system is allowed to equilibrate for 30 min, after which a background spectrum is recorded. Subsequently, a series of scans (99 scans per measurement, ∼1 min per measurement, data spacing: 0.482 cm^–1^) is initiated at the onset of the carburization profile (25 to 700 °C, ramp rate: 10 °C/min). The IR vibrational bands are assigned based on comparisons with published literature, as summarized in Table S2. The integrated peak areas for the respective IR absorption bands are quantified as illustrated in Figure S21.

### 4.9 Reactor Studies

To evaluate the catalytic performance of *ex situ* synthesized catalysts, temperature-programmed reactions are conducted over the 200–300 °C (TPR200–300 °C) at a ramp rate of 15 °C h^–1^. The concentrations of reactants and products are analyzed using an in-line Agilent Technologies 7890B gas chromatograph (GC) equipped with a flame ionization detector (FID) and a thermal conductivity detector (TCD). A 20 mg catalyst sample is loaded into a horizontally mounted stainless-steel reactor (OD: 6.35 mm, ID: 4.57 mm). Before reaction, all *ex situ* synthesized catalyst samples undergo prereduction at 350 °C (10 °C/min) and 50 psi under pure H_2_ (40 mL min^–1^, 120,000 mL h^–1^ g^–1^) for 2 h. Following the prereduction step, the reactor is cooled to 200 °C (3.33 °C min^–1^) under H_2_. Once the reactor stabilized at 200 °C, a reactant gas mixture of H_2_:CO_2_:Ar (6:2:1) with a H_2_:CO_2_ of 3:1 (total flow rate: 45 mL min^–1^, GHSV: 135,000 mL h^–1^ g^–1^) is introduced, and the system is pressurized to 300 psi. The reactor is held at 200 °C for 1 h to equilibrate, after which the temperature is ramped at 0.25 °C min^–1^ to the desired reaction temperature, with GC injections recorded throughout the process. Ar is used as the internal standard, and the concentration of each gas-phase species is calibrated by correlating its peak area to a known calibration standard. CO STY is evaluated by the following equation.

$$\mathrm{CO}\;\mathrm{STY}=\frac{\mathrm{GHSV}\times S_{\mathrm{CO}}\times x_{{\mathrm{CO}}_{2}}\times {\mathrm{MW}}_{\mathrm{CO}}}{\left(1+\frac{H_{2}+\mathrm{Ar}}{{\mathrm{CO}}_{2}}\right)\times V_{m}}\times \frac{1}{W_{m}}$$

where GHSV is the gas hourly space velocity (L hr^–1^ g_cat_^–1^), *S*_CO_ is the CO selectivity, *x*_CO_2__ is the CO_2_ conversion, MW_CO_ is the molar mass of CO (g/mol), *V*_m_ is the molar volume at STP (L/mol), and *W*_m_ is the weight fraction of W metal in the catalyst.

To evaluate the stability of supported W_2_C catalysts for CO_2_ hydrogenation, a 100-h time-on-stream (TOS) reactor study is conducted under isothermal conditions at 300 °C, using a 20 mg sample of W_2_C/SiO_2_ (15W/SiO_2_-835 °C), following the same experimental setup as the TPR measurements. GC injections are recorded after 1 h of equilibration and continuously monitored over 100 h to assess catalyst stability. TPR-200-300 °C measurements on *in situ* synthesized catalysts were conducted directly after carburization within the reactor. Following carburization, the reactor was cooled to 200 °C under the synthesis gas mixture, after which the gas was switched to a reactant mixture of H_2_:CO_2_:Ar (6:2:1), corresponding to a H_2_:CO_2_ ratio of 3:1, and the system was pressurized to 300 psig. The catalyst was then allowed to equilibrate for 1 h prior to initiating TPR from 200 to 300 °C at a ramp rate of 15 °C h^–1^.

### 4.10 Surface Area Analysis as a Function of the Degree of Passivation

TCD signal analysis during the initial 30 min of passivation following carburization under 1% O_2_/N_2_ is performed using a Micromeritics AutoChem II analyzer equipped with a thermal conductivity detector (TCD). A 100 mg sample of calcined supported catalyst is loaded into a quartz U-tube reactor. The sample is subjected to temperature-programmed carburization (TPC) at 835 °C under conditions similar to those described in Catalyst Preparation. A total flow rate of 28 mL min^–1^ is applied to achieve the same gas velocity of 10 cm min^–1^ as the typical TPC conditions. The TCD signal is recorded continuously during the passivation step. The TCD signal area observed during the first 30 min of passivation is assumed to be proportional to the total active carbide surface area of the catalyst.

## Supplementary Material



## Notes

Additional experimental details, figures, and tables including *d*-spacing calculations, Rietveld refinements, hypothesized carburization model, crystallite size determination, XRD, TEM, and XPS analyses, carburization profiles, reaction results, DRIFTS studies, and IR peak assignments (PDF)
