# Supplementary material for: Achieving Phase Control of Polymorphic Tungsten Carbide Catalysts
Source: ACS Catal. 2026 Jan 5;16(2):1542–55. doi: 10.1021/acscatal.5c07774 (PMC12813974; doi:10.1021/acscatal.5c07774)
Supplement: Supplementary file 1 [file cs5c07774_si_001.pdf]

Supporting Information

Achieving Phase Control of Polymorphic Tungsten Carbide Catalysts

Sinhara M.H.D. Perera, Eva Ciuffetelli, and Marc D. Porosoff*

Department of Chemical and Sustainability Engineering, University of Rochester, Rochester, NY 14627, USA

*Corresponding author. E-mail: marc.porosoff@rochester.edu

Table of Contents

1. Theoretical calculation of d-spacings 1

2. Reitveld refinement 2

3. Hypothesized carburization model for TPC 2

4. Determination of W_2C crystallite size 3

5. Figures 4

6. Tables 19

7. References 20

1. Theoretical calculation of d-spacings

$$\frac{1}{d^2} = \frac{h^2}{a^2 sin^2\beta} + \frac{k^2}{b^2} + \frac{l^2}{c^2 sin^2\beta} + \frac{2hl \cos\beta}{ac\ sin^2\beta} \quad \text{(S1)}$$

The interplanar spacing (d) for a monoclinic crystal system can be determined using the above equation,[1] where h, k, l are the Miller indices of the crystal plane, a, b, c are the lattice constants, and β is the angle between the a and c axes. The lattice parameters for monoclinic WO_3 with the space group $P2_1/n$ are a = 7.3 Å, b = 7.53 Å, c = 7.68 Å, and β = 90.54°. Using these parameters, the theoretically calculated d-spacings for the (220) and (400) planes are determined to be 2.62 Å and 1.82 Å, respectively.

2. Rietveld refinement

Rietveld refinement of the XRD patterns is performed using the ***FullProf*** software package. The refinement was applied to the 2θ range of 25-60°, where all major diffraction peaks corresponding to both W_2C and WC phases are present. Identical refinement parameters are used for all patterns as described below. The refinements were sequentially conducted for the following parameters: scale factor, background, instrumental zero, lattice parameters, preferred orientation, shape parameters, FWHM parameters.

Peak shape:	Pseudo Voigt
Background mode:	6 coefficient polynomial function
Cycle of refinement:	20
Refinement weighting model:	Least squares
Reflections ordering:	Only at first cycle
Relaxation factors:	Atomic:1, Anisotropic:1, Profile:1, Global:1

3. Hypothesized carburization model for TPC

The proposed mechanistic model for TPC under initial reduction-carburization conditions is outlined in **Table S3**. According to the temperature profile, WO_3 undergoes complete reduction to metallic W before reaching 490 °C. At this temperature, CH_4 dissociates in the absence of lattice oxygen (L_O), generating graphitic carbon, which subsequently reacts with W to form WC_{1-x} ($0 \leq x < 1$).

The generalized reaction model is presented below, representing overall reactions derived from the elementary steps detailed in **Table S3**. The initial carburization rate is governed by the rate-limiting step, which involves the surface reaction generating graphitic carbon and the subsequent diffusion of carbon into the solid matrix. Assuming carburization occurs under carbon-limited conditions at mild temperatures, the overall rate is dictated by the surface reaction producing graphitic carbon. The reduction and carburization processes initiate at the particle surface and progressively advance toward the particle core. As depicted in **Figure 4**, at time t=t, a carburized outer shell ($r_i - r_t$) encases an unreacted tungsten (W) core. The instantaneous carburization shell, Δr, represents the reaction front where unreacted W interacts with carbon to form tungsten carbide.

T < 320 °C

$$WO_{y(s)} + \alpha\, CH_{4(g)} \rightarrow WO_{y-\alpha(s)} + \alpha\, CO + \; 2\alpha\, H_{2(g)}$$

320 °C < T < 490 °C

$$WO_{y(s)} + \beta\, H_{2(g)} \rightarrow W_{y-\beta(s)} + \; \beta\, H_2O_{(g)}$$

$$WO_{y(s)} + \alpha\, CH_{4(g)} \rightarrow WO_{y-\alpha(s)} + \alpha\, CO + \; 2\alpha\, H_{2(g)}$$

$$WO_{y(s)} + \alpha\, CH_{4(g)} \rightarrow WO_{y-2\alpha(s)} + \alpha\, CO_2 + \; 2\alpha\, H_{2(g)}$$

T > 490 °C

$$CH_{4(g)} \leftrightarrow C_{(s)} + 2\, H_{2(g)}$$

$$W_{(s)} + (1-x)\, C_{(s)} \;\rightarrow WC_{1-x(s)}$$

4. Determination of W_2C crystallite size

The crystallite size was determined from XRD using the Scherrer equation (Equation S2). The W_2C crystallite size was calculated from the highest-intensity W_2C reflection, corresponding to the (121) plane.

$$D = \frac{K\lambda}{\beta\, cos\theta} \qquad \text{(S2)}$$

Where D is the crystallite size (nm), K is the shape factor (taken as 0.9), β is the full width at half-maximum (FWHM, radians), λ is X-ray wavelength (1.54184 Å), and θ is the Bragg angle. The FWHM was determined after correcting for instrumental broadening via Rietveld refinement using the FullProf software.

5. Figures

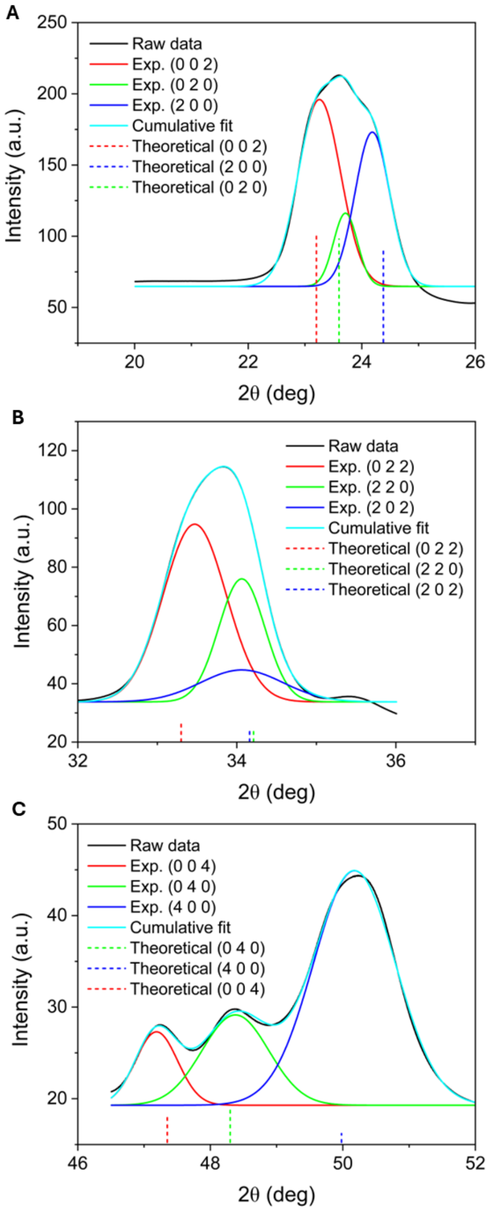

Figure S1: Detailed comparison between the deconvoluted experimental XRD peaks of WO_3 and the theoretical XRD peaks corresponding to monoclinic WO_3 with space group $P2_1/n$.

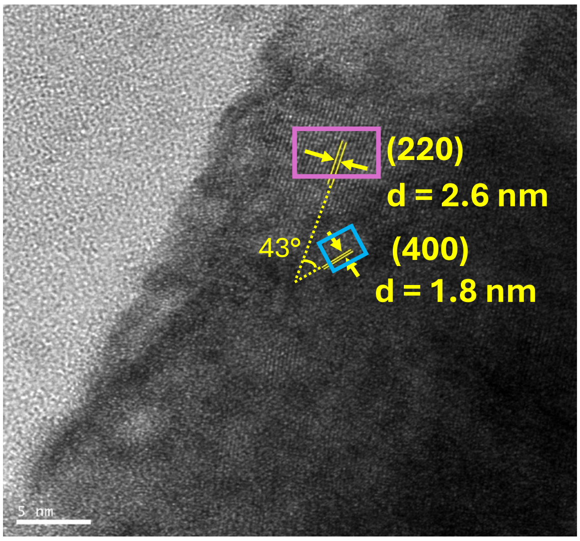

Figure S2: HR-TEM image for 15W/SiO_2-cal, showing well-defined lattice fringes of WO_3 corresponding to (220) and (400) planes.

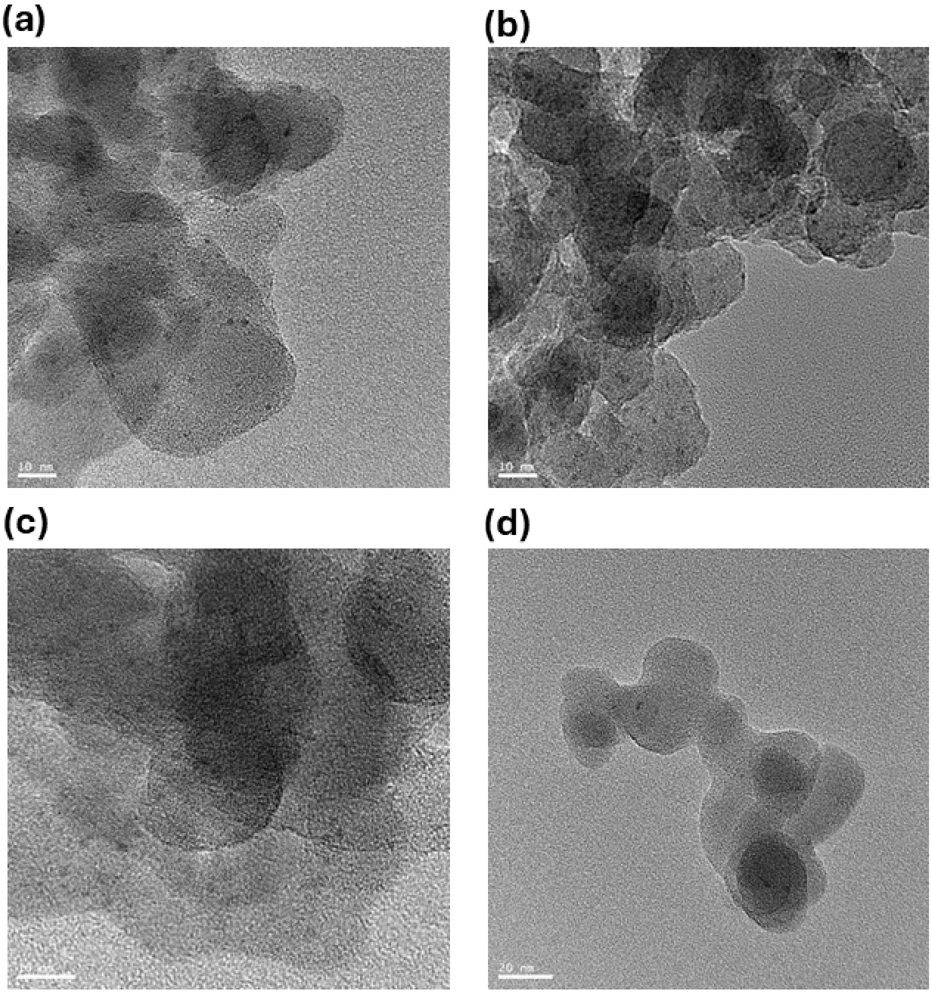

Figure S3: TEM images of 15W/SiO2-cal with scale bars of **(a-b)** 10 nm and **(c-d)** 20 nm.

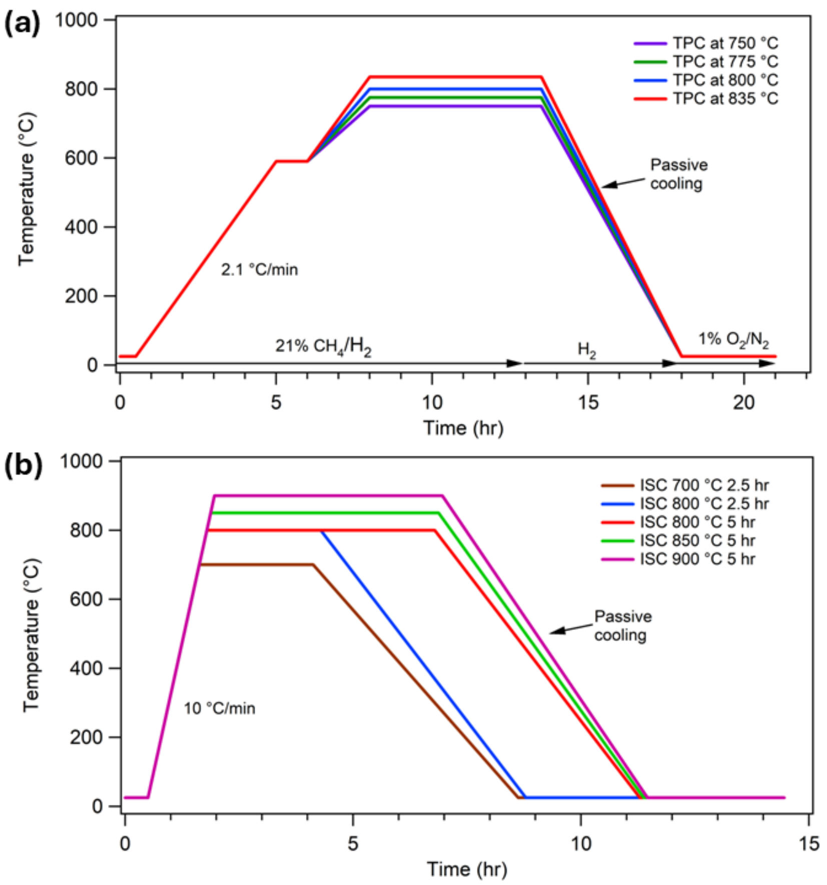

Figure S4: Carburization temperature profiles. **(a)** Temperature-programmed carburization (TPC). **(b)** Isothermal carburization (ISC).

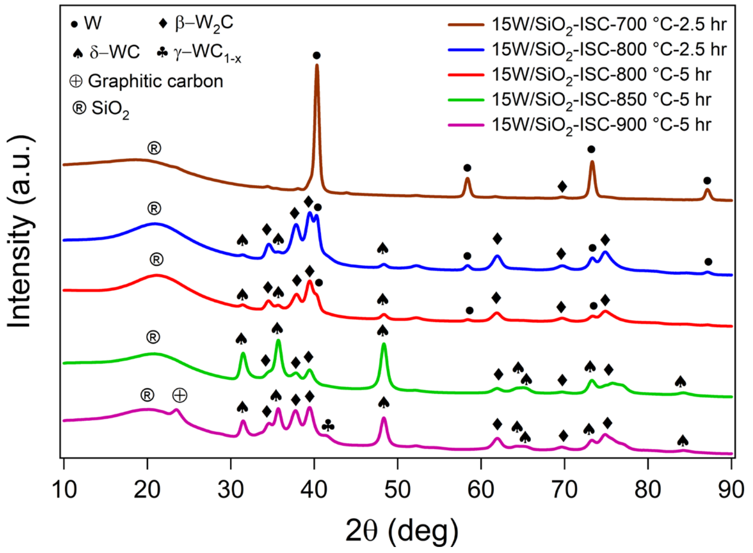

Figure S5: XRD patterns of carburized 15W/SiO_2 as a function of temperature via isothermal carburization (ISC).

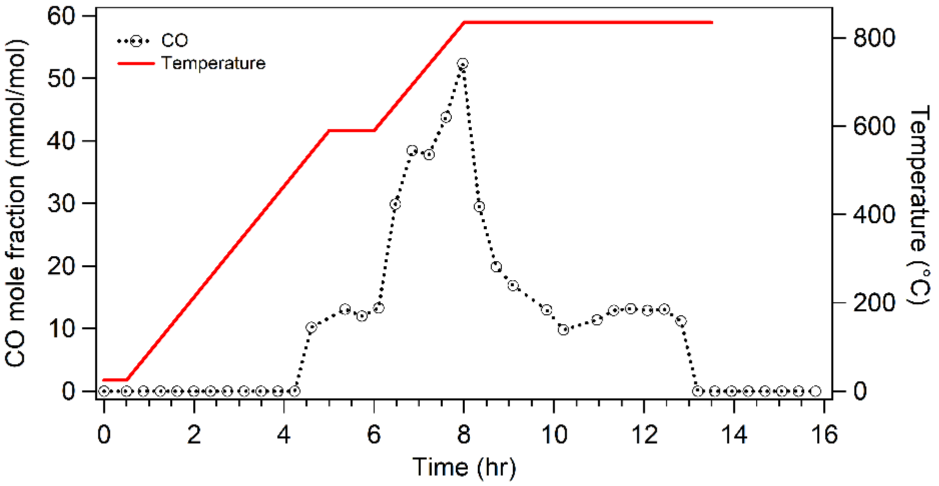

Figure S6: Gas chromatography (GC) analysis of gaseous product evolution during temperature-programmed carburization (TPC) of $15W/SiO_2$-cal at 835 °C.

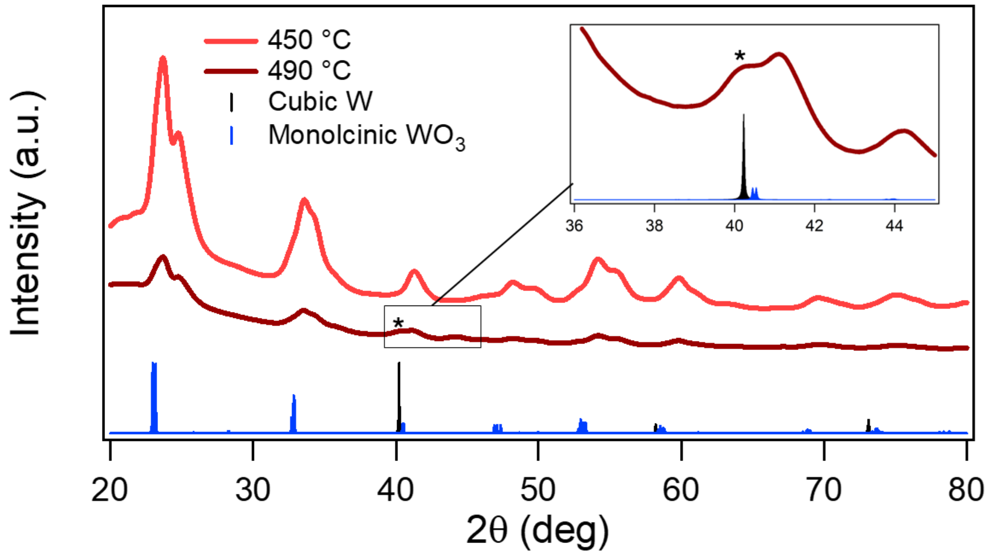

Figure S7: Quasi-in situ XRD analysis of supported WO_3 ($15W/SiO_2$) via TPC. The XRD pattern at 450 °C indicates that the bulk of the particles remains as WO_3. At 490 °C, the significant reduction in WO_3 peak intensities, together with the emergence of metallic W reflections (marked by * in the zoomed-in inset), indicates substantial reduction prior to the onset of carburization at 490 °C.

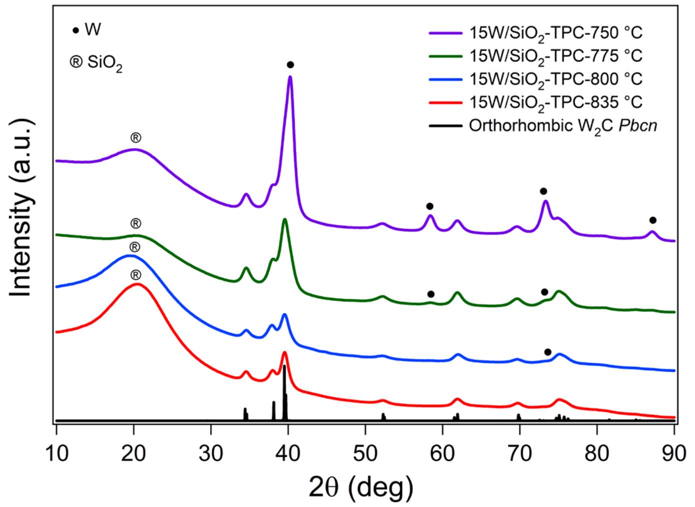

Figure S8: XRD patterns of carburized 15W/SiO_2 as a function of temperature via temperature-programmed carburization (TPC).

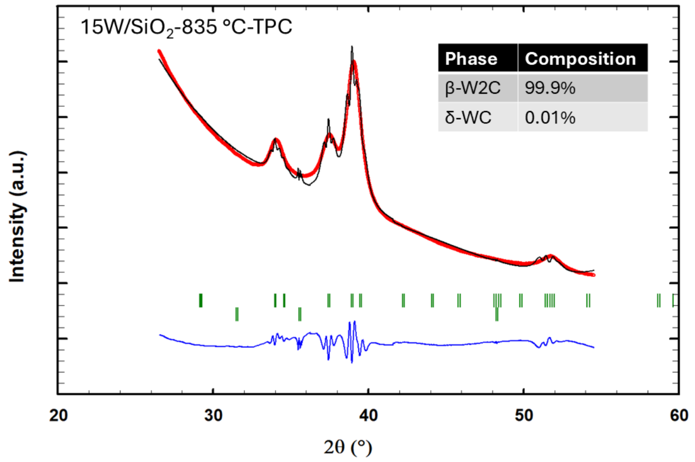

Figure S9: Rietveld-refined XRD pattern of 15W/SiO_2 at 835 °C via TPC, indicating 99.9 wt.% β-W_2C. The observed (red), calculated (black), and residuals (blue) are presented for each sample, with Bragg positions indicated by vertical green markers.

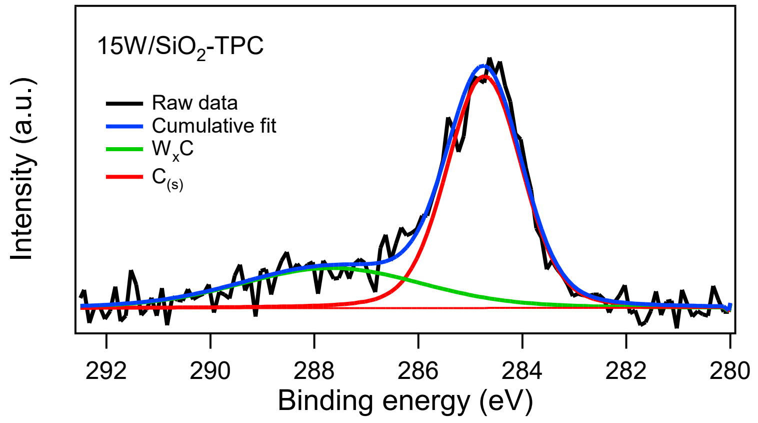

Figure S10: C 1s XPS spectra of 15W/SiO_2-TPC-835 °C.

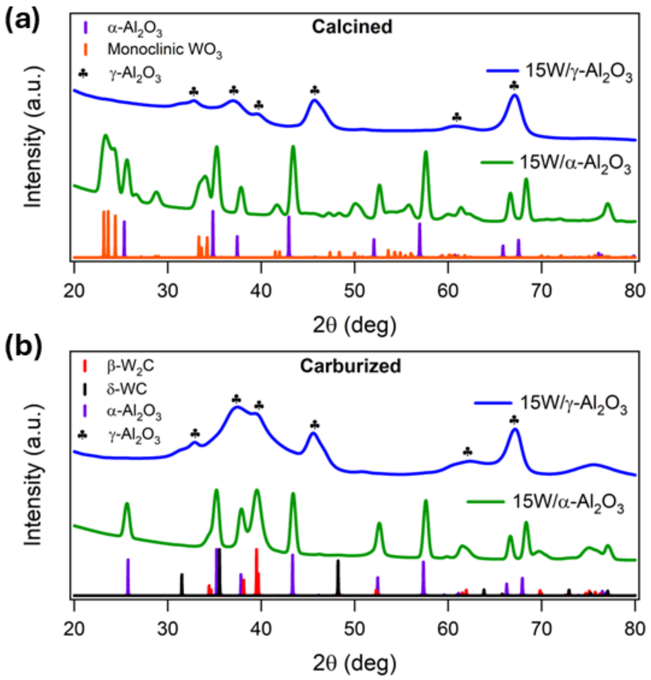

Figure S11: XRD patterns of **(a)** calcined WO_x supported on the α and γ phases of Al_2O_3, and **(b)** the respective carburized catalysts.

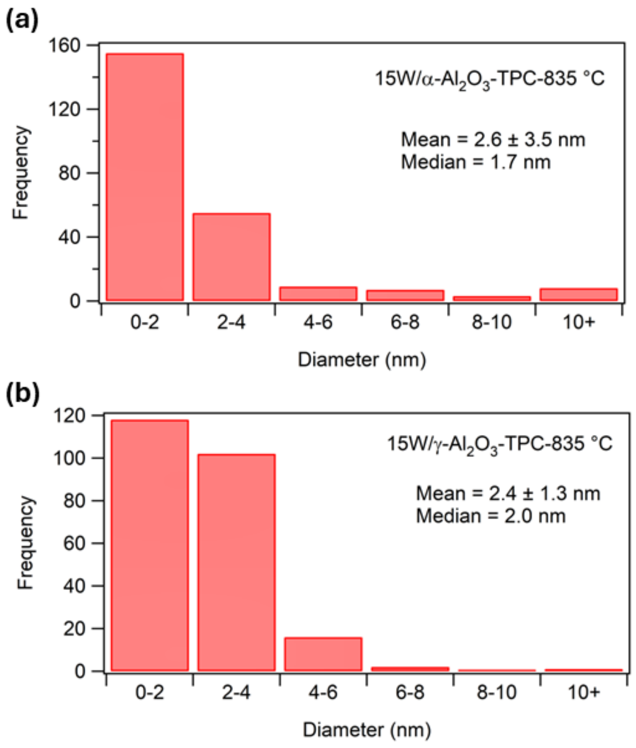

Figure S12: TEM-based particle size distribution analysis for **(a)** 15W/α-Al_2O_3-TPC-835 °C and **(b)**15W/γ-Al_2O_3-TPC-835 °C.

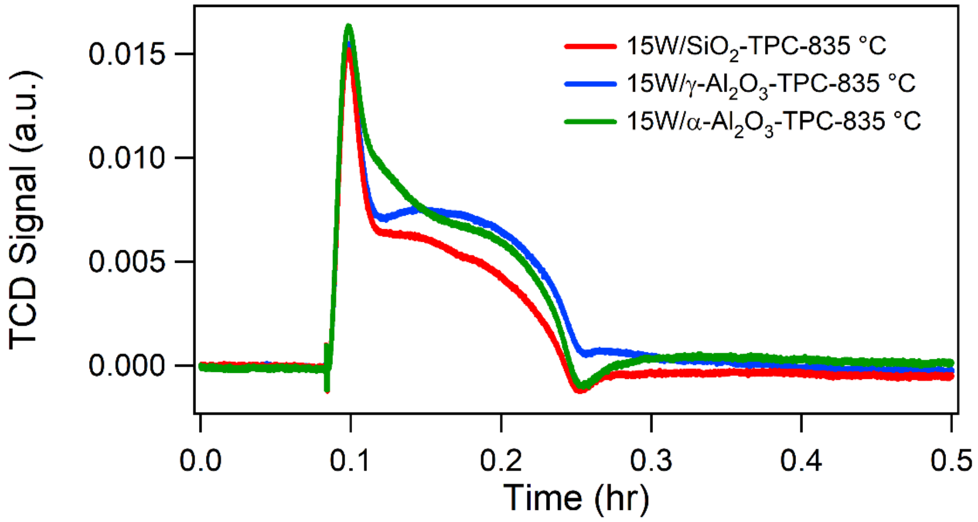

Figure S13: TCD signal during post-carburization passivation under 1% O_2/N_2 at 35 °C

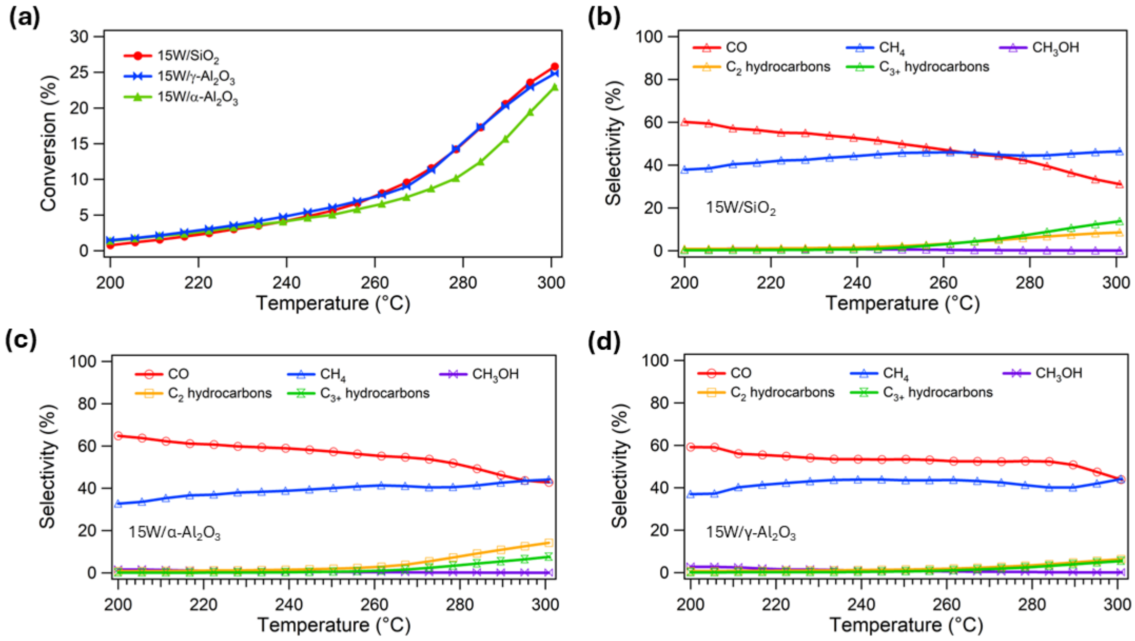

Figure S14: Comparison of RWGS performance for W_xC supported on SiO_2, α-Al_2O_3, and γ-Al_2O_3. **(a)** CO_2 conversion; Product selectivity for **(b)** 15W/SiO_2-TPC-835 °C, **(c)** 15W/α-Al_2O_3-TPC-835 °C, and **(d)** 15W/γ-Al_2O_3-TPC-835 °C. Reaction conditions: T = 200-300 °C, 0.25 °C min^{-1}, H_2:CO_2 = 3:1, P = 300 psig, GHSV = 135,000 mL h^{-1} g^{-1}. All samples are pre-reduced at 350 °C for 2 hours prior to reactions. The carbon balance for all reactions is within 100% ± 1%.

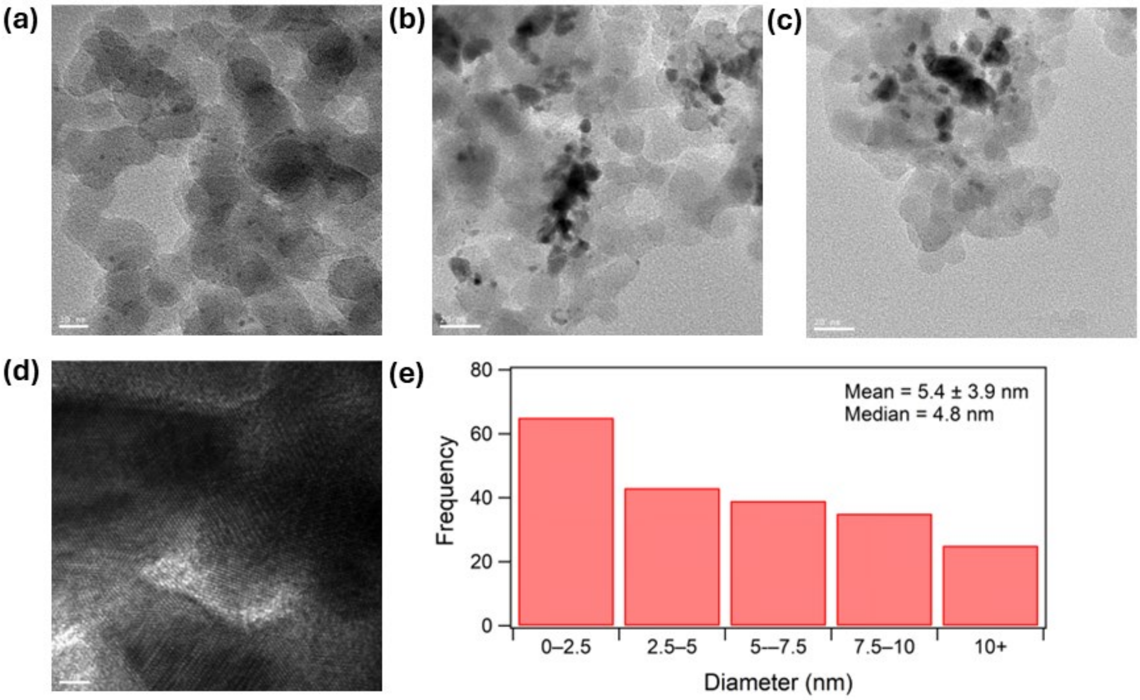

Figure S15: TEM analysis of 50W/SiO_2-TPC-835 °C. **(a)** TEM image at 10 nm scale. **(b-c)** TEM images at 20 nm scale. **(d)** High-resolution TEM (HR-TEM) image at 2 nm scale. **(e)** Particle size distribution histogram.

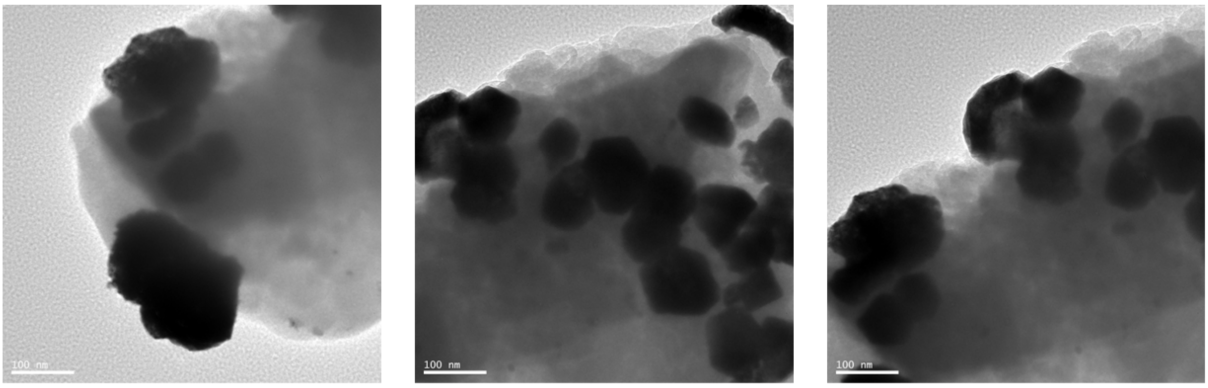

Figure S16: TEM images of unsupported WC carburized via TPC at 835 °C for 5 h. The darker particles are identified as W_xC, whereas the lighter, diffuse regions surrounding the W_xC particles are attributed to carbon that is deposited during carburization, as evidenced by XPS analysis of supported W_2C (Figure S10).

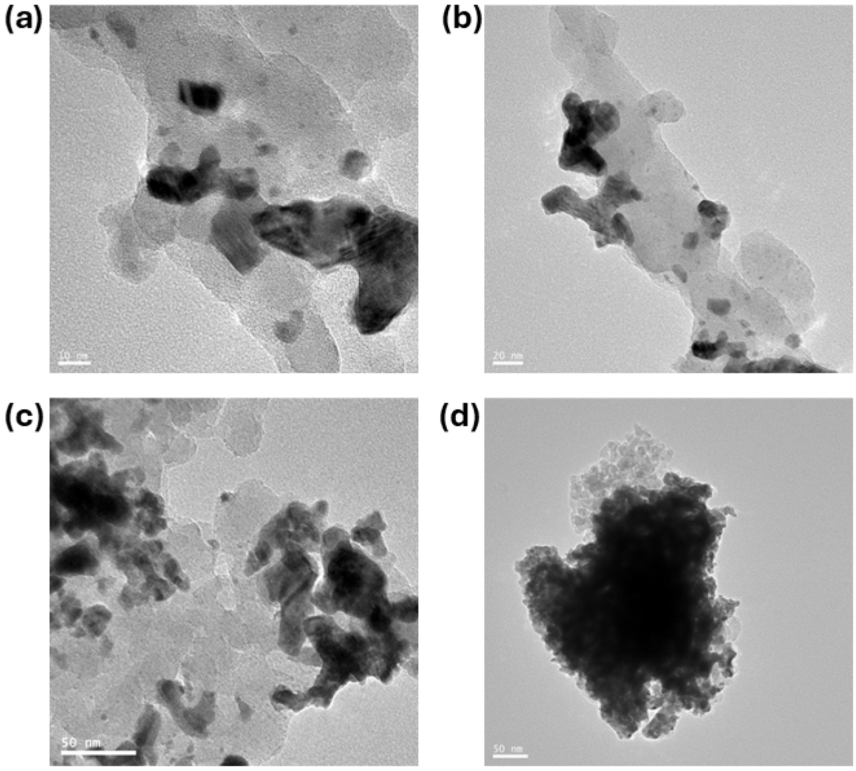

Figure S17: TEM images of support-removed W_xC carburized via TPC at 835 °C for 5 h. **(a)** 10 nm, **(b)** 20 nm, **(c-d)** 50 nm scale bar. The darker particles are identified as W_xC, whereas the lighter, diffuse regions surrounding the W_xC particles are attributed to carbon that is deposited during carburization, as evidenced by XPS analysis of supported W_2C (Figure S10)

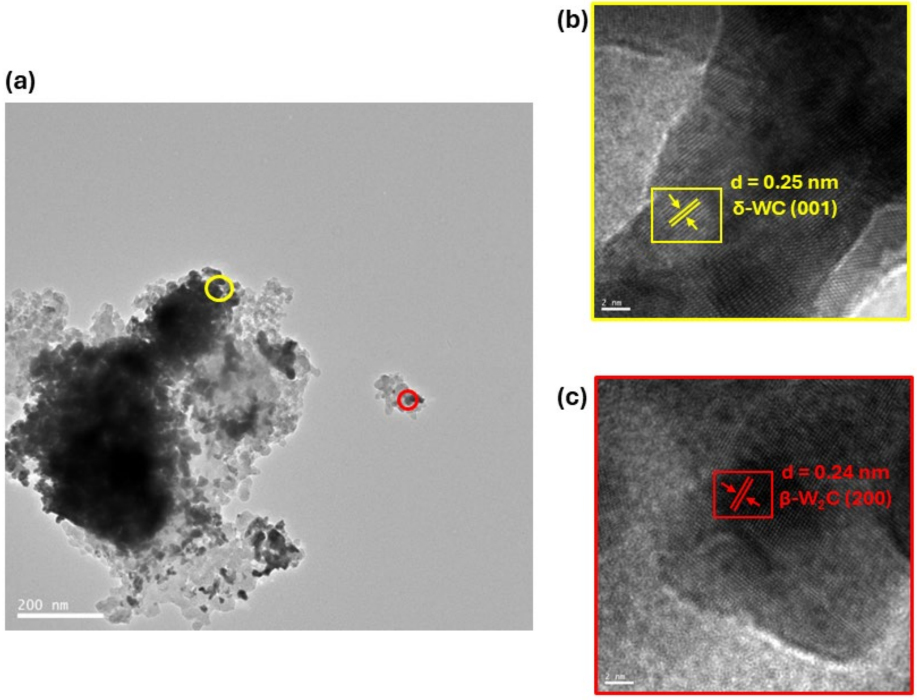

Figure S18: HR-TEM images of support-removed W_xC. **(a)** TEM image showing sub-10 nm particles and larger aggregates. **(b-c)** HRTEM images of yellow circle in (a), identifying the (001) plane of δ-WC within a larger aggregate and the red circle in (a), identifying the (200) plane of β-W_2C.

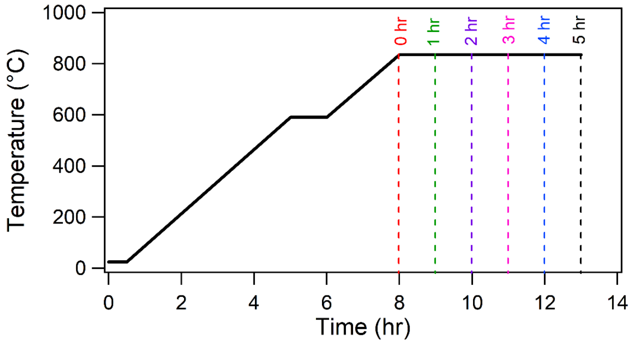

Figure S19: Temperature profile used in the quasi *in situ* XRD analysis, with dashed lines indicating points where carburization is interrupted for XRD measurements.

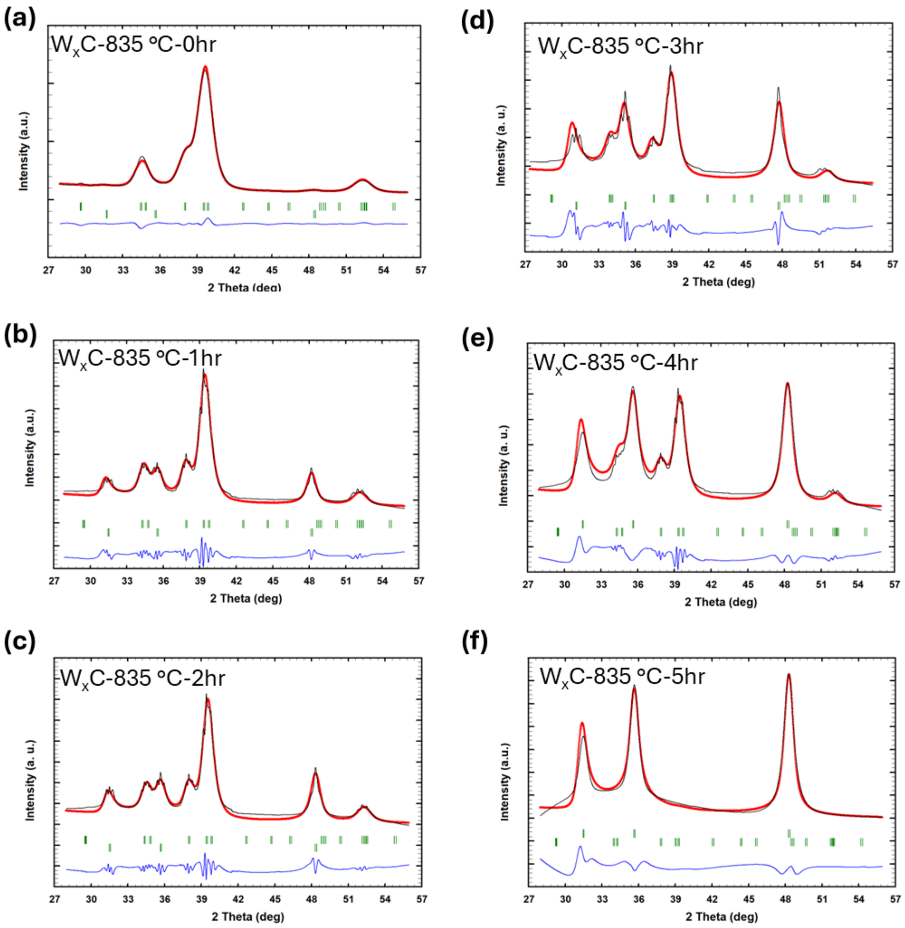

Figure S20: **(a-f)** Rietveld-refined XRD patterns of WO_3 carburized via TPC at 835 °C for varying durations (0-5 hours). The observed (red), calculated (black), and residuals (blue) curves are presented for each sample, with Bragg positions indicated by vertical green markers.

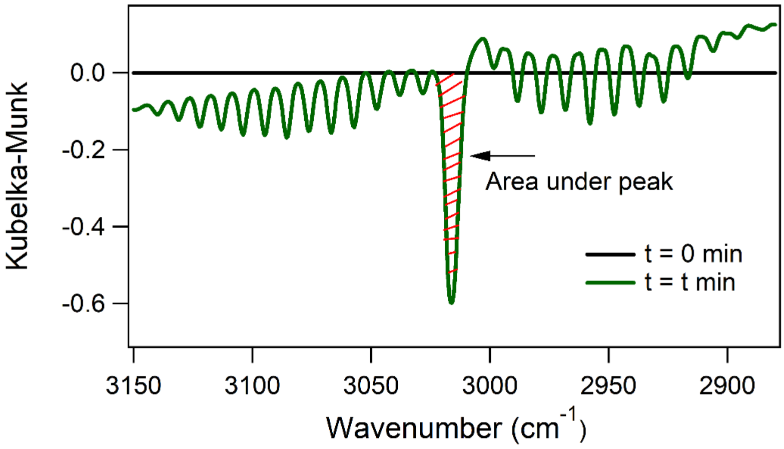

Figure S21: The integrated peak area of the CH_4 symmetric stretching vibration mode (ν_3) at ~3016 cm^{-1} is obtained via *in situ* DRIFTS during the TPC of 15W/SiO_2-cal under a 2:5 v/v ratio of 21% CH_4 in H_2 and Ar, with a total flow rate of 14 mL min^{-1}. The CH_4 ν_3 peak area is quantified relative to the background spectrum recorded under the same reaction atmosphere at the start of the experiment (t = 0 min).

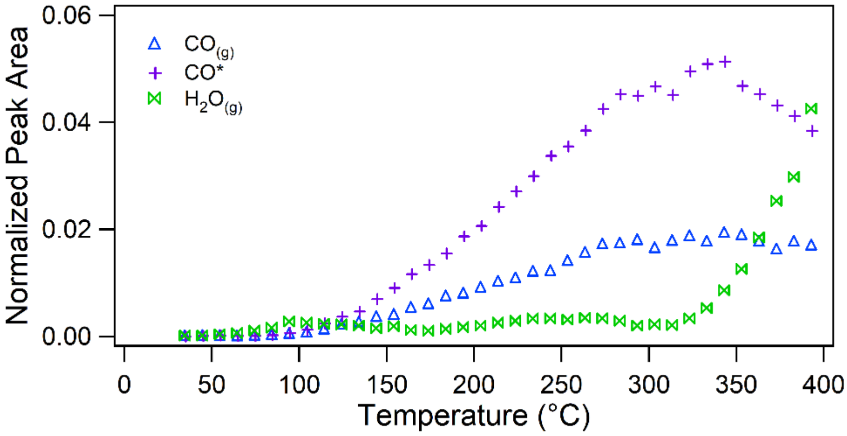

Figure S22: Evolution of CO and H_2O during carburization below 400 °C, showing the normalized peak areas of the respective IR bands as a function of temperature.

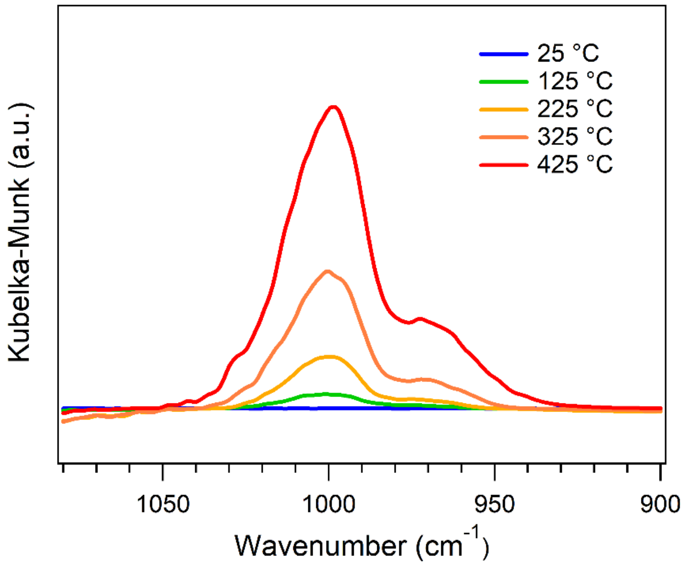

Figure S23: Time-resolved DRIFTS spectra showing the evolution of HyWO$_x$ species during the initial stages of WO_3 carburization below 400 °C.

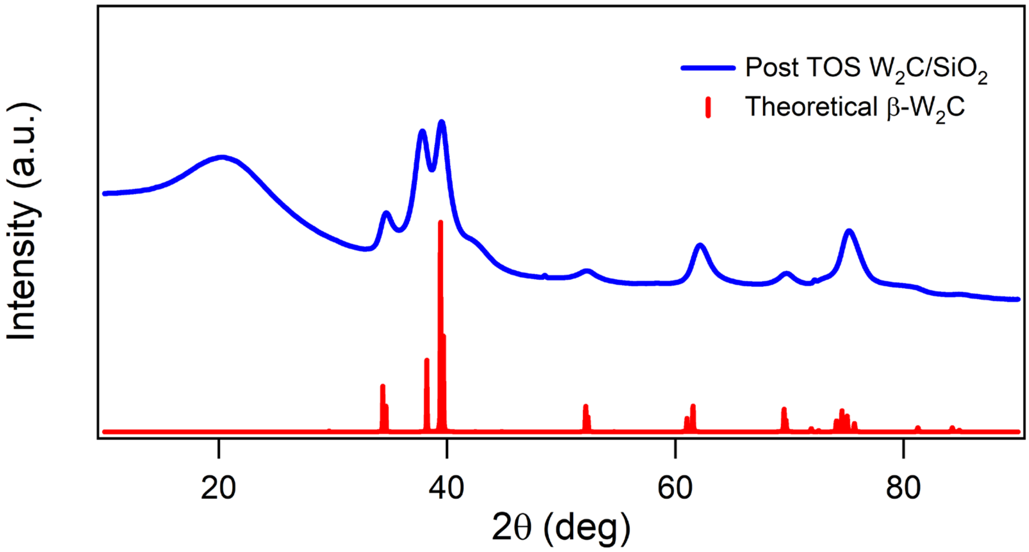

Figure S24: XRD pattern of post time-on-stream W_2C/SiO_2 (ex-situ 15W/SiO_2-835 °C) catalyst, indicating phase stability under reaction conditions.

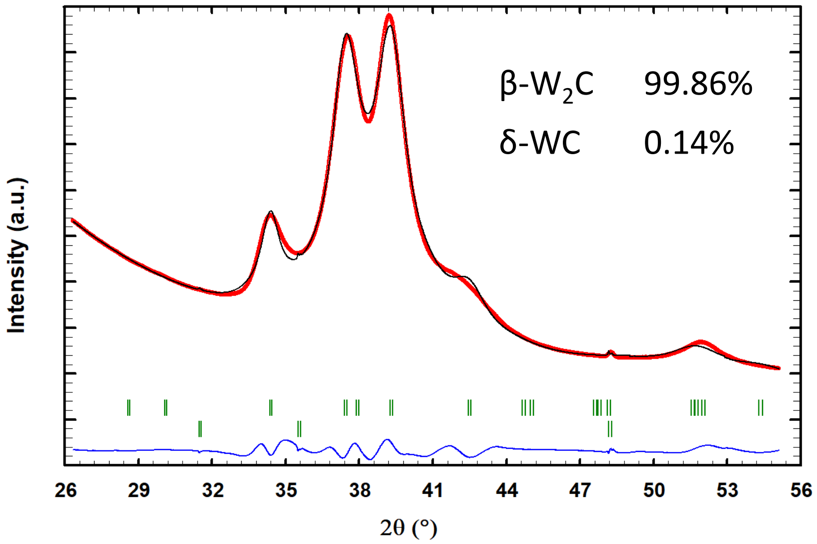

Figure S25: Rietveld-refined XRD pattern of post time-on-stream W_2C/SiO_2 (ex-situ 15W/SiO_2-835 °C) catalyst, which shows 99.86 wt.% β-W_2C.

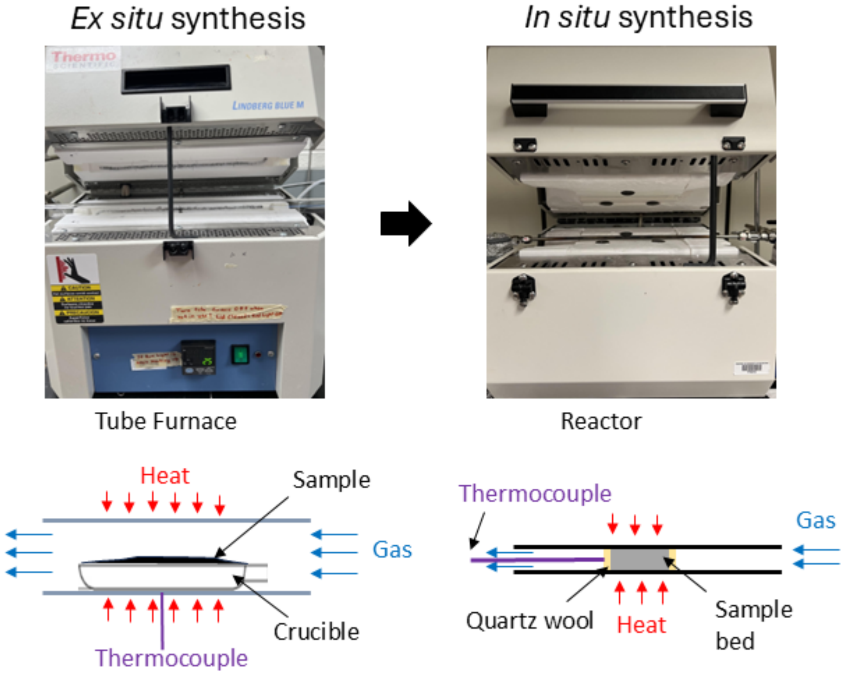

Figure S26: Schematic illustration showing the differences between *ex situ* synthesis of W_xC in a tube furnace and *in situ* synthesis within the reactor.

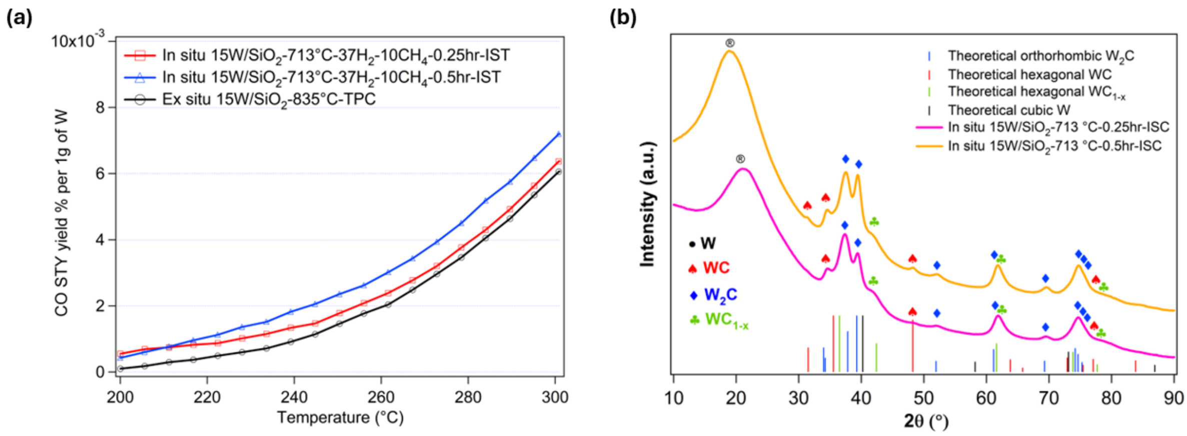

Figure S27: Structure-function relationships of *in situ* synthesized W_xC via ISC at different carburization durations, synthesis gas flow rate is 47 mL min^{-1} (21 vol% CH_4 in H_2) and carburization temperature is 713 °C. **(a)** CO STY during CO_2 hydrogenation, TPR 200-300 °C, H_2:CO_2 = 3:1, P = 300 psig, GHSV = 27,000 mL h^{-1} g^{-1}. The *ex situ* synthesized catalyst is pre-reduced at 350 °C for 2 hours prior to reaction. The carbon balance for all reactions is within ±1% of 100%. **(b)** Post-reaction XRD patterns of 15W/SiO_2-T°C-ISC, where T stands for carburization temperature.

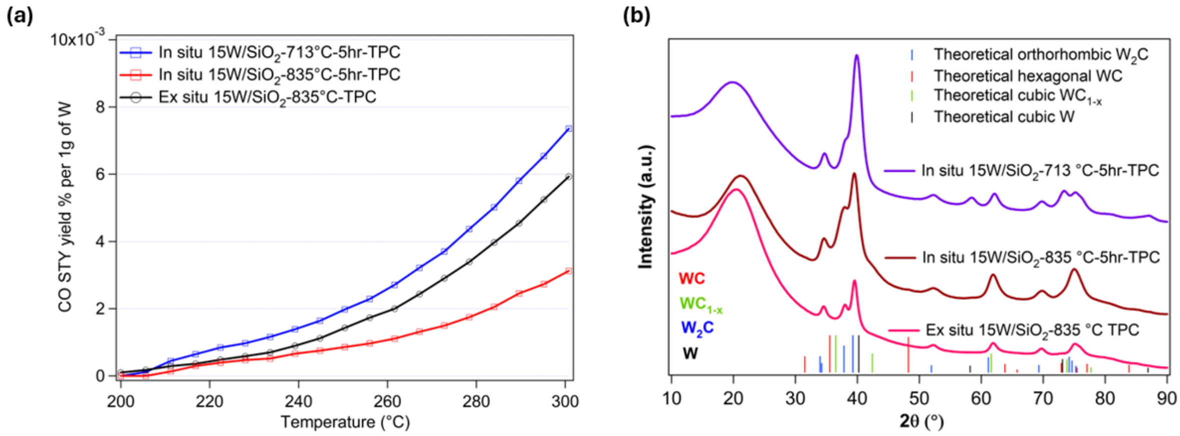

Figure S28: Structure-function relationships of *in situ* synthesized W_xC via TPC at different carburization temperatures, synthesis gas flow rate is 47 mL min^{-1} (21 vol% CH_4 in H_2) and carburization duration is 5 hr. **(a)** CO STY during CO_2 hydrogenation, TPR-200-300 °C, H_2:CO_2 = 3:1, P = 300 psig, GHSV = 27,000 mL h^{-1} g^{-1}. The *ex situ* synthesized catalyst is pre-reduced at 350 °C for 2 hours prior to reaction. The carbon balance for all reactions is within ±1% of 100%. **(b)** Post-reaction XRD patterns of 15W/SiO_2-T°C-TPC, where T stands for carburization temperature.

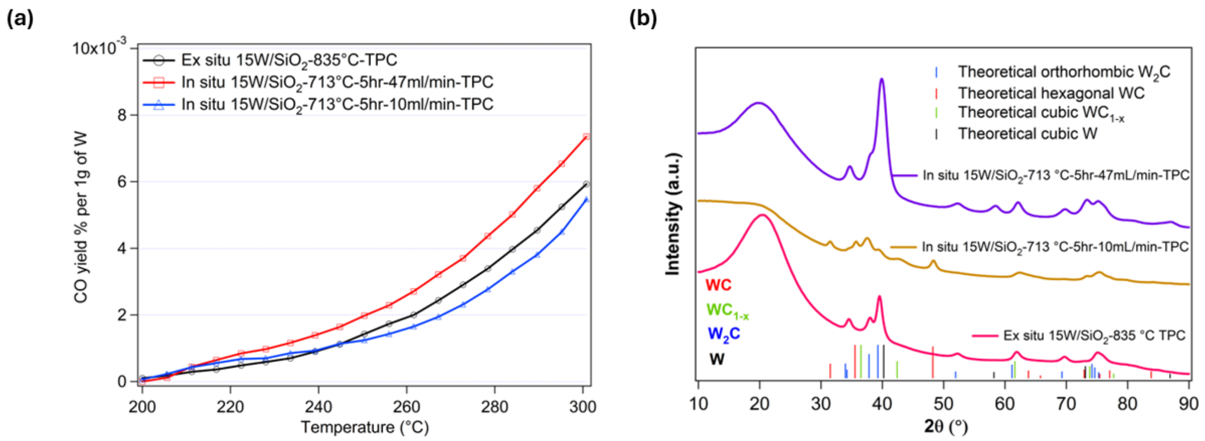

Figure S29: Structure-function relationships of *in situ* synthesized W_xC via TPC at different gas residence times, carburization temperature and duration are respectively 713 °C and 5 hr. **(a)** CO STY during CO_2 hydrogenation, TPR 200-300 °C, H_2:CO_2 = 3:1, P = 300 psig, GHSV = 27,000 mL h^{-1} g^{-1}. The *ex situ* synthesized catalyst is pre-reduced at 350 °C for 2 hours prior to reaction. The carbon balance for all reactions is within ±1% of 100%. **(b)** Post-reaction XRD patterns of 15W/SiO_2-L-713°C-5hr-TPC, where L stands for feed gas flowrate.

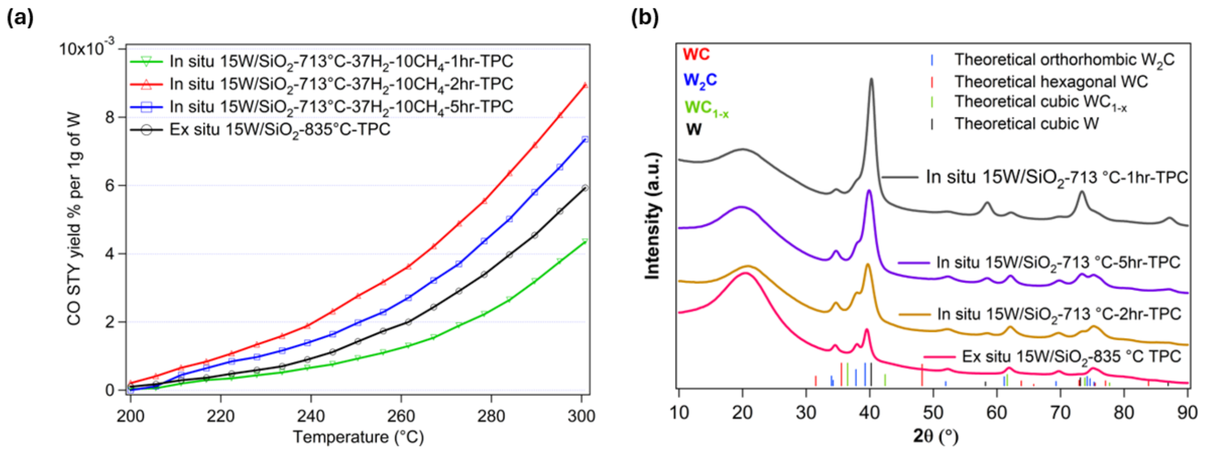

Figure S30: Structure-function relationships of *in situ* synthesized W_xC via TPC at different carburization durations, synthesis gas flow rate is 47 mL min^{-1} (21 vol% CH_4 in H_2) and carburization temperature is 713 °C. **(a)** CO STY during CO_2 hydrogenation, TPR 200-300 °C, H_2:CO_2 = 3:1, P = 300 psig, GHSV = 27,000 mL h^{-1} g^{-1}. The *ex situ* synthesized catalyst is pre-reduced at 350 °C for 2 hours prior to reaction. The carbon balance for all reactions is within ±1% of 100%. **(b)** Post-reaction XRD patterns of 15W/SiO_2-713°C-t-TPC, where t stands for carburization duration.

6. Tables

Table S1: Weight fractions of phases obtained from Rietveld refinements for unsupported WO_3 carburized via TPC at 835 °C for 0-5 hour durations.

**Sample**	**W_2C wt.%**	**WC wt.%**
W_xC-835 °C-0hr	99.7 ± 3.5	0.27 ± 0.1
W_xC-835 °C-1hr	77.3 ± 2.0	22.7 ± 0.8
W_xC-835 °C-2hr	66.7 ± 2.1	33.3 ± 1.1
W_xC-835 °C-3hr	49.4 ± 1.8	50.6 ± 1.2
W_xC-835 °C-4hr	33.6 ± 1.3	66.4 ± 1.4
W_xC-835 °C-5hr	0.0 ± 0.0	100.0 ± 1.6

Table S2: IR peak assignments of gas-phase products and surface-adsorbed species during temperature programmed carburization (TPC) of WO_3/SiO_2

IR absorption band	Band center for this study (cm^{-1})	Band center from literature (cm^{-1})
CH_4 ν_3	~3016	~3016 [2]
CH_4 ν_4	~1304	~1306 [2]
CO_2 ν_3	~2340	~2348 [2]
CO ν	~2140	~2143 [2]
*CO ν	~1945	~1800 on WO_3 [3]<br>~2000 (Linear atop) and ~1900 (bridging) on Ru[4]
H_2O δ	1380–1560	1300–1600 [5]
*OOCH ν_3	~1690	~1650 on WO_3 [3]<br>~1594 on Au-ZnO/TiO_2[6]<br>~1676 on Cu[7]
*OCH_3 δ	~1355	~1363 on WO_3 [3]
*OCH_3 $\nu_{(C-O)}$	~1127	~1065 on V_2O_5/TiO_2 [8]
*OCH_2 δ	~1250	~1251 on WO_3 [3]<br>~1280 on Au-ZnO/TiO_2 [6]

Table S3: Proposed reaction mechanism for temperature programmed carburization (TPC)

**T < 320 °C (excess *H)**	**320 °C < T < 490 °C (excess *OH)**
$CH_{4(g)}$ + L_O → *OCH_3 + *H<br>H_2 → 2 *H<br>*H + L_O → *OH	H_2 + L_O → *H + *OH<br>$CH_{4(g)}$ + L_O → *OCH_3 + *H<br>*H + *OH → H_2O<br>*H + L_O → *OH
*OCH_3+ *H → *OCH_2 + H_2	*OCH_3 + *OH → *OCH2 + H_2O
*OCH_2 + *OH → *OOCH + H_2	*OCH_2 + *OH → *OOCH + H_2
*OOCH → *CO + *OH	*OOCH → *CO + *OH<br>*OOCH + *OH → $CO_{2(g)}$ + $H_2O_{(g)}$
*CO → $CO_{(g)}$	*CO → $CO_{(g)}$

7. References

(1) Kelly, A.; Knowles, K. M. Appendix 3: Interplanar Spacings and Interplanar Angles. In *Crystallography and Crystal Defects*, 2012; pp 469-472.

(2) Wasylenko, W.; Frei, H. Direct observation of the kinetically relevant site of CO hydrogenation on supported Ru catalyst at 700 K by time-resolved FT-IR spectroscopy. *Phys Chem Chem Phys* **2007**, *9* (40), 5497-5502. DOI: 10.1039/b706689a.

(3) Fan, Y.; Jiang, Y.; Lin, H.; Li, J.; Xie, Y.; Chen, A.; Li, S.; Han, D.; Niu, L.; Tang, Z. Insight into selectivity of photocatalytic methane oxidation to formaldehyde on tungsten trioxide. *Nat Commun* **2024**, *15* (1), 4679. DOI: 10.1038/s41467-024-49138-8.

(4) Liu, J.; Hibbitts, D.; Iglesia, E. Dense CO Adlayers as Enablers of CO Hydrogenation Turnovers on Ru Surfaces. *J Am Chem Soc* **2017**, *139* (34), 11789-11802. DOI: 10.1021/jacs.7b04606.

(5) Seki, T.; Chiang, K. Y.; Yu, C. C.; Yu, X.; Okuno, M.; Hunger, J.; Nagata, Y.; Bonn, M. The Bending Mode of Water: A Powerful Probe for Hydrogen Bond Structure of Aqueous Systems. *J Phys Chem Lett* **2020**, *11* (19), 8459-8469. DOI: 10.1021/acs.jpclett.0c01259.

(6) Song, S.; Song, H.; Li, L.; Wang, S.; Chu, W.; Peng, K.; Meng, X.; Wang, Q.; Deng, B.; Liu, Q.; et al. A selective Au-ZnO/TiO2 hybrid photocatalyst for oxidative coupling of methane to ethane with dioxygen. *Nature Catalysis* **2021**, *4* (12), 1032-1042. DOI: 10.1038/s41929-021-00708-9.

(7) Gomes, J. R. B.; Gomes, J. A. N. F. Adsorption of the formate species on copper surfaces: a DFT study. *Surface Science* **1999**, *432* (3), 279-290. DOI: 10.1016/s0039-6028(99)00605-6.

(8) Burcham, L. J.; Briand, L. E.; Wachs, I. E. Quantification of Active Sites for the Determination of Methanol Oxidation Turn-over Frequencies Using Methanol Chemisorption and in Situ Infrared Techniques. 1. Supported Metal Oxide Catalysts. *Langmuir* **2001**, *17* (20), 6164-6174. DOI: 10.1021/la010009u.
